# Loss of synovial tissue macrophage homeostasis precedes rheumatoid arthritis clinical onset

**DOI:** 10.1126/sciadv.adj1252

**Published:** 2024-09-25

**Authors:** Megan M. Hanlon, Conor M. Smith, Mary Canavan, Nuno G. B. Neto, Qingxuan Song, Myles J. Lewis, Aoife M. O’Rourke, Orla Tynan, Brianne E. Barker, Phil Gallagher, Ronan Mullan, Conor Hurson, Barry Moran, Michael G. Monaghan, Costantino Pitzalis, Jean M. Fletcher, Sunil Nagpal, Douglas J. Veale, Ursula Fearon

**Affiliations:** ^1^ Molecular Rheumatology, School of Medicine, Trinity College Dublin, Dublin, Ireland.; ^2^ Centre for Arthritis and Rheumatic Diseases, St. Vincent's University Hospital, University College Dublin, Dublin, Ireland.; ^3^ School of Biochemistry and Immunology, Trinity College Dublin, Dublin, Ireland.; ^4^ Translational Immunopathology, School of Biochemistry and Immunology and School of Medicine, Trinity College Dublin, Dublin, Ireland.; ^5^ Department of Mechanical and Manufacturing Engineering, Trinity Biomedical Sciences Institute, Dublin, Ireland.; ^6^ Immunology and Discovery Sciences, Janssen Research and Development, Spring House, PA, USA.; ^7^ Centre for Experimental Medicine and Rheumatology, William Harvey Research Institute, Queen Mary University of London and Barts NIHR BRC and Barts Health NHS Trust, London, UK.; ^8^ Department of Rheumatology, Adelaide and Meath Hospital, Dublin, Ireland.; ^9^ Department of Orthopaedics, St. Vincent’s University Hospital, Dublin, Ireland.; ^10^ Department of Biomedical Sciences, Humanitas University and Humanitas Research Hospital, Milan, Italy.; ^11^ School of Medicine, Trinity Biomedical Sciences Institute, Trinity College Dublin, Dublin, Ireland.

## Abstract

This study performed an in-depth investigation into the myeloid cellular landscape in the synovium of patients with rheumatoid arthritis (RA), “individuals at risk” of RA, and healthy controls (HC). Flow cytometric analysis demonstrated the presence of a CD40-expressing CD206^+^CD163^+^ macrophage population dominating the inflamed RA synovium, associated with disease activity and treatment response. In-depth RNA sequencing and metabolic analysis demonstrated that this macrophage population is transcriptionally distinct, displaying unique inflammatory and tissue-resident gene signatures, has a stable bioenergetic profile, and regulates stromal cell responses. Single-cell RNA sequencing profiling of 67,908 RA and HC synovial tissue cells identified nine transcriptionally distinct macrophage clusters. IL-1B^+^CCL20^+^ and SPP1^+^MT2A^+^ are the principal macrophage clusters in RA synovium, displaying heightened CD40 gene expression, capable of shaping stromal cell responses, and are importantly enriched before disease onset. Combined, these findings identify the presence of an early pathogenic myeloid signature that shapes the RA joint microenvironment and represents a unique opportunity for early diagnosis and therapeutic intervention.

## Teaser

In-depth analysis of myeloid cells residing within the RA joint reveals a unique early signature associated with disease activity.

## INTRODUCTION

Rheumatoid arthritis (RA) is a progressive autoimmune disease characterized by synovial inflammation, hyperplasia, and structural damage to cartilage and bone (*1*). It affects 1% of the population, reduces mobility and quality of life, and is associated with substantial comorbidities including atherosclerosis, diabetes, cardiovascular disease, and obesity (*2*–*4*). A significant proportion of patient’s are nonresponders to current therapeutic targets, and it is currently impossible to predict who will develop severe, erosive disease and who will respond to treatment. Therefore, better understanding of the disease at the site of inflammation will allow the development of new treatment strategies or predictive biomarkers.

Macrophages are pivotal players in joint destruction, with increased numbers of sub-lining macrophages being a hallmark of disease activity and response to treatment in RA (*5*–*9*). Recent research has revealed a diversity of macrophage phenotypes in health and disease, in both function and origin (*10*). This has further emphasized the need to explore and characterize the phenotype and ontogeny of macrophage populations in RA. Immunohistology analysis of RA synovial tissue suggests that macrophages residing in the synovial lining layer differ from those in the sub-lining layer (*11*). Mature resident macrophages are abundant in the intimal lining layer, displaying an interleukin-10 (IL-10) phenotype (*12*). In contrast, the synovial sub-lining has a more heterogeneous phenotype, displaying a mixture of both inflammatory M1-like and resolving M2-like markers, possibly due to active infiltration of monocyte-derived macrophages from the periphery (*12*, *13*).

A conceptual revolution has occurred in recent years, challenging our understanding of the origin of macrophages, demonstrating that many macrophages are tissue-derived during embryonic development (*14*–*18*). Many tissue-resident macrophages arise from embryonic precursors before birth independent of hematopoiesis and subsequently self-sustain their numbers throughout adulthood (*19*–*21*). Evidence suggests that the relative contribution of infiltrating versus resident macrophages in establishing a pool of mature macrophages differs from one tissue to another (*14*, *15*, *22*–*24*). Moreover, the prevailing dogma that activated macrophages exist as one of two phenotypical states, M1 or M2, while useful, fails to reflect the remarkable plasticity and diversity of these cells in human disease. Macrophages in vivo are subject to a plethora of stimuli, capable of changing their phenotype in response to environmental cues, such that the real-time phenotype most likely does not fit this rigid binary nomenclature (*25*, *26*). Evidence now suggests that this system should be extended to encompass a wide spectrum of macrophage activation states, the characteristics and ontogeny of which remain largely unknown (*27*, *28*).

A recent study has reported in a mouse model of arthritis that CX_3_CR1^+^ macrophages in the synovial lining layer form an unusual protective barrier-like layer to shield the joint from inflammation associated with arthritis (*29*). Another recent study described the presence of a specific subset of tissue macrophages capable of re-establishing joint homeostasis in RA (*30*). This study described differential enrichment of two general subpopulations of macrophages, MerTK^+^ and MerTK^−^, with risk of flare determined by the ratio of these synovial tissue macrophages (*30*). This highlights the plasticity of macrophages in adapting to the needs of their microenvironment. Local macrophage differentiation is determined by tissue-specific cues with tissue imprinting a dominant factor in shaping the phenotype and function of macrophages (*31*–*34*).

Undeniably, the role of macrophages in the pathogenesis of RA is widely recognized, yet this has been largely based on in vitro monocyte-derived macrophage analysis, synovial histology, or animal studies (*35*–*37*). Recent advances in transcriptomic analysis have improved our understanding of macrophage biology in RA synovium with some studies investigating human synovial tissue macrophages using methods such as single-cell RNA sequencing (scRNA-seq) and mass cytometry (*30*, *38*). Recently, it has been established that circulating autoantibodies can precede clinical onset of symptoms in patients with RA and so studying those “at risk” of developing disease may provide important clues in understanding RA disease pathogenesis (*39*). However, the relative contribution of synovial tissue macrophages in the evolution of RA, especially before disease, has yet to be fully elucidated.

Therefore, in this study, we used multiparameter flow cytometry, bulk RNA-seq and scRNA-seq, and noninvasive fluorescent lifetime imaging microscopy (FLIM) metabolic imaging and functional analysis to fully explore the spectrum of macrophage activation states residing within the synovium of patients with RA, individuals at risk of RA (IAR), and healthy controls and determine their role in driving RA pathogenesis.

## RESULTS

### CD206^+^CD163^+^ macrophages are the dominant macrophage subset in RA synovial tissue

**Fig. 1.**
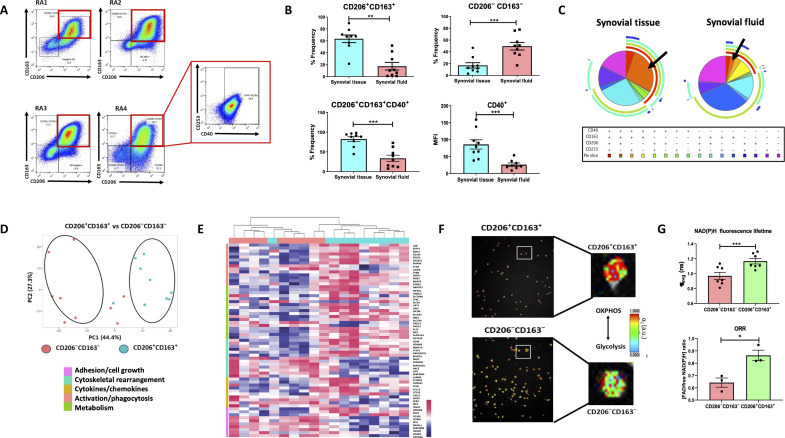
Synovial tissue macrophage phenotypic characterization. (**A**) Representative flow cytometric dot plots of four independent RA synovial tissue samples demonstrating the frequency of CD206^+^CD163^+^ macrophages and coexpression of CD40. (**B**) Dot plots indicating percentage frequency of CD206^+^CD163^+^, CD206^−^CD163^−^, and CD206^+^CD163^+^CD40^+^ macrophages and median fluorescence intensity (MFI) of CD40 on CD206^+^CD163^+^ macrophages in RA synovial tissue (*n* = 9) in comparison to synovial fluid (*n* = 9). Data are presented as mean ± SEM with each symbol representing a different sample. Statistical analysis using Mann-Whitney *U* test, *P < 0.05, **P < 0.01, ***P < 0.005, significantly different from synovial tissue. (**C**) Visual representation of multidimensional flow cytometric data. SPICE analysis was performed for the identification of distinct macrophage subsets in an average of RA synovial tissue (*n* = 9) and fluid (*n* = 6). Each pie segment indicates the different combinations of marker expression as denoted by the legend below. The surrounding pie arcs indicate the specific macrophage markers produced by each pie segment. High-quality RNA was isolated from sorted CD206^+^CD163^+^ and CD206^−^CD163^−^ synovial tissue macrophages and bulk RNA-seq was performed. (**D**) PCA was performed on the total dataset of RA synovial tissue sorted CD206^+^CD163^+^ and CD206^−^CD163^−^ macrophage subsets (*n* = 9). (**E**) Hierarchical clustered heatmap displaying DEGs involved in adhesion/cell growth, cytoskeletal rearrangement, cytokine/chemokines, macrophage markers/phagocytosis, and metabolism in RA synovial tissue CD206^+^CD163^+^ macrophages compared to CD206^−^CD163^−^ macrophages (*n* = 9). (**F**) Representative multiphoton microscopy FLIM analysis of flow sorted RA CD206^+^CD163^+^ and CD206^−^CD163^−^ synovial tissue macrophages. Representative FLIM images whereby a red/green cell is predominantly using OXPHOS, while a blue cell that indicates glycolysis is being used as the main energy source. (**G**) Summary of macrophage emission lifetime (τ_avg_) (*n* = 7) and optical redox ratio (ORR) (*n* = 3). Data expressed as mean ± SEM using Wilcoxon signed rank or paired *t* test, *P < 0.05, ***P < 0.005 significantly different from each other.

Within the inflamed microenvironment of the joint in vivo, macrophages are exposed to a plethora of stimuli; therefore, we examined both M1/M2-like and non-M1/M2 macrophage phenotypes within RA synovial tissue. Patient demographics are outlined in Table 1. RA synovial tissue single-cell suspensions and synovial fluid mononuclear cells (SFMCs) were stained using a panel of human macrophage polarization–associated cell surface markers to assess the phenotype and frequency of macrophages (Fig. 1, A to C, and fig. S1A). The frequency and median fluorescence intensity (MFI) of pan macrophage markers CD68 and CD64 were significantly increased in RA synovial tissue compared to SFMC, indicating that a greater number of macrophages reside in synovial tissue in comparison to the synovial fluid (fig. S1B, *P* < 0.05). Analysis of specific macrophage markers identified a spectrum of activation states rather than the classic paradigm of M1 and M2 (Fig. 1). On the basis of this spectrum, we identified a dominant population of synovial tissue macrophages in the RA inflamed joint expressing high levels of CD206 and CD163, markers typical of an M2-like phenotype (Fig. 1A and fig. S1C). This double-positive population also expressed high levels of the activation marker CD40 (Fig. 1, A and B, and fig. S1C).

**Table 1.** Patient demographics for RA synovial tissue. Clinical information of patients with RA for synovial tissue samples included in this study (*n* = 32). Rheumatoid factor (RF), anti-citrullinated protein antibodies (ACPA), C-reactive protein (CRP), disease activity score-28 (DAS28), disease duration, and mediation at the time of arthroscopy are shown. Values reflect either the group average or the range in each group with the given criteria.

Characteristics	Mean	Percentage or range
Age (years)	54.8	24–80
Gender, female	10	31%
RF positive	16	50%
ACPA positive	19	59.3%
Disease duration (years)	9.2	<1–41
DAS28	3.9	1.68–7.74
CRP (mg/liter)	25.5	<1–156.7
On MTX	12	37.50%
Treatment naïve	10	31%

A marked increased expression of CD206^+^CD163^+^ macrophages residing in RA synovial tissue compared to fluid was observed as indicated by representative flow plots and accompanying frequencyquantification (Fig. 1B and fig. S1D). In contrast, the double-negative CD206^−^CD163^−^ subset of macrophages is significantly enriched in synovial fluid in comparison to tissue (Fig. 1B). Next, we used the Simplified Presentation of Incredibly Complex Evaluations (SPICE) algorithm. SPICE analysis facilitated further visual confirmation of site-specific macrophage phenotypes (Fig. 1C), where we demonstrate, as indicated by the deep red pie segment, that the CD206^+^CD163^+^CD40^+^ macrophage subset is markedly greater in RA synovial tissue compared to fluid (Fig. 1C). The specific enrichment of CD206^+^CD163^+^CD40^+^ macrophages in RA synovial tissue compared to fluid is further highlighted and confirmed by representative flow plots, histograms, and relative proportion bar graph quantification (Fig. 1B and fig. S1, D and E).

### CD206^+^CD163^+^ macrophages are transcriptionally distinct with unique metabolic preferences

To further explore the phenotype of CD40-expressing CD206^+^CD163^+^ RA synovial macrophages, we profiled the transcriptome of this cell population using RNA-seq analysis. High-quality mRNA was isolated from sorted RA synovial tissue/fluid macrophages and paired RA monocyte-derived M1- and M2-like phenotype, and bulk RNA-seq was performed. Although M1- and M2-like macrophages are not reflective of true macrophage phenotypes in vivo, they are useful as extremes of the spectrum of macrophage activation states. Here, we investigated whether synovial tissue CD206^+^CD163^+^ macrophages more closely resemble a pro-inflammatory M1-like phenotype or immunoregulatory M2-like phenotype. Figure S2A presents the gating strategy used to identify and sort pure macrophage populations from RA synovial tissue and fluid samples. Patient demographics are summarized in Table 2. Table 3 summarizes the individual yields of CD206^+^CD163^+^ and CD206^−^CD163^−^ populations from each digested biopsy sample.

**Table 2.** Patient demographics sorted RA synovial tissue biopsies used for bulk RNA-seq. Clinical information of patients with RA for synovial tissue samples included in this study (*n* = 9). RF, ACPA, CRP, DAS28, disease duration, and mediation at the time of arthroscopy are shown. Values reflect either the group average or the range in each group with the given criteria.

Characteristics	Average	Percentage or range
Age (years)	50.7	31–72
Female	6	66.7%
RF positive	4	44%
ACPA positive	3	33.3%
Disease duration (years)	11.2	<1–35
DAS28	4.2	2.4–6.6
CRP	14	1–31
On MTX	2	25%
On biologic	4	50%
Treatment naïve	3	33%

**Table 3.** Yields of sorted macrophages from digested RA synovial biopsy samples. The table indicates the yields of specific macrophage populations FACS-sorted from RA synovial tissue biopsies (*n* = 9).

	Starting cell count	CD206^+^CD163^+^	CD206^−^CD163^−^
	3 × 10^6^	9247	16,145
	3 × 10^6^	108,000	60,000
	6 × 10^6^	823,000	72,000
	6 × 10^6^	5500	6500
	4 × 10^6^	149,000	79,000
	3.3 × 10^6^	2619	1124
	2 × 10^6^	5500	4400
	3.5 × 10^6^	2500	5500
	4 × 10^6^	130,000	70,000
Average	3.6 × 10^6^	139,374	35,963.22222

Principal components analysis (PCA) of the total gene expression dataset demonstrates that CD206^+^CD163^+^ macrophages cluster separately from RA CD206^−^CD163^−^ macrophages (Fig. 1D). Further interrogation identified differences in genes associated with adhesion/cell growth, cytoskeletal rearrangement, inflammatory mediators, phagocytosis, and cellular metabolism (Fig. 1E). Inflammatory genes such as *CCL8, CCL13, CCL18, CXCL3*, and *MMP14* are all significantly enriched in CD206^+^CD163^+^ synovial macrophages while adhesion genes such as *ICAM3* and *SELL* are reciprocally enriched in CD206^−^CD163^−^ macrophages (Fig. 1E). Together, these data indicate distinct transcriptional profiles between RA synovial tissue CD206^+^CD163^+^ and CD206^−^CD163^−^ macrophages.

Given the significant role of metabolic rewiring in shaping RA macrophage activation states (*40*, *41*), we investigated the bioenergetic profiles of CD206^+^CD163^+^ synovial tissue macrophages. RNA-seq revealed differential expression of metabolism-associated genes between CD206^+^CD163^+^ and CD206^−^CD163^−^ macrophages (Fig. 1E). To date, no study has assessed the live metabolic competence of human RA synovial tissue macrophages, mainly due to the difficulty in isolating sufficient cells for standard metabolic assays. However, here we use FLIM analysis of sorted RA synovial tissue macrophages (*n* = 7), revealing notably consistent differences in metabolic preferences between CD206^+^CD163^+^ and CD206^−^CD163^−^ macrophages. CD206^+^CD163^+^ synovial tissue macrophages used oxidative phosphorylation as their main source of energy, while the CD206^−^CD163^−^ macrophages preferentially use glycolysis as indicated by the NAD(P)H fluorescence lifetime and representative FLIM images (Fig. 1, F and G, *P* < 0.05). The optical redox ratio that examines the ratio between FAD and NADH is significantly increased in CD206^+^CD163^+^ macrophages, further supporting a reliance on oxidative metabolism (Fig. 1G, *P* < 0.05).

### CD206^+^CD163^+^CD40^+^ RA synovial tissue macrophages are associated with increased disease activity

**Fig. 2.**
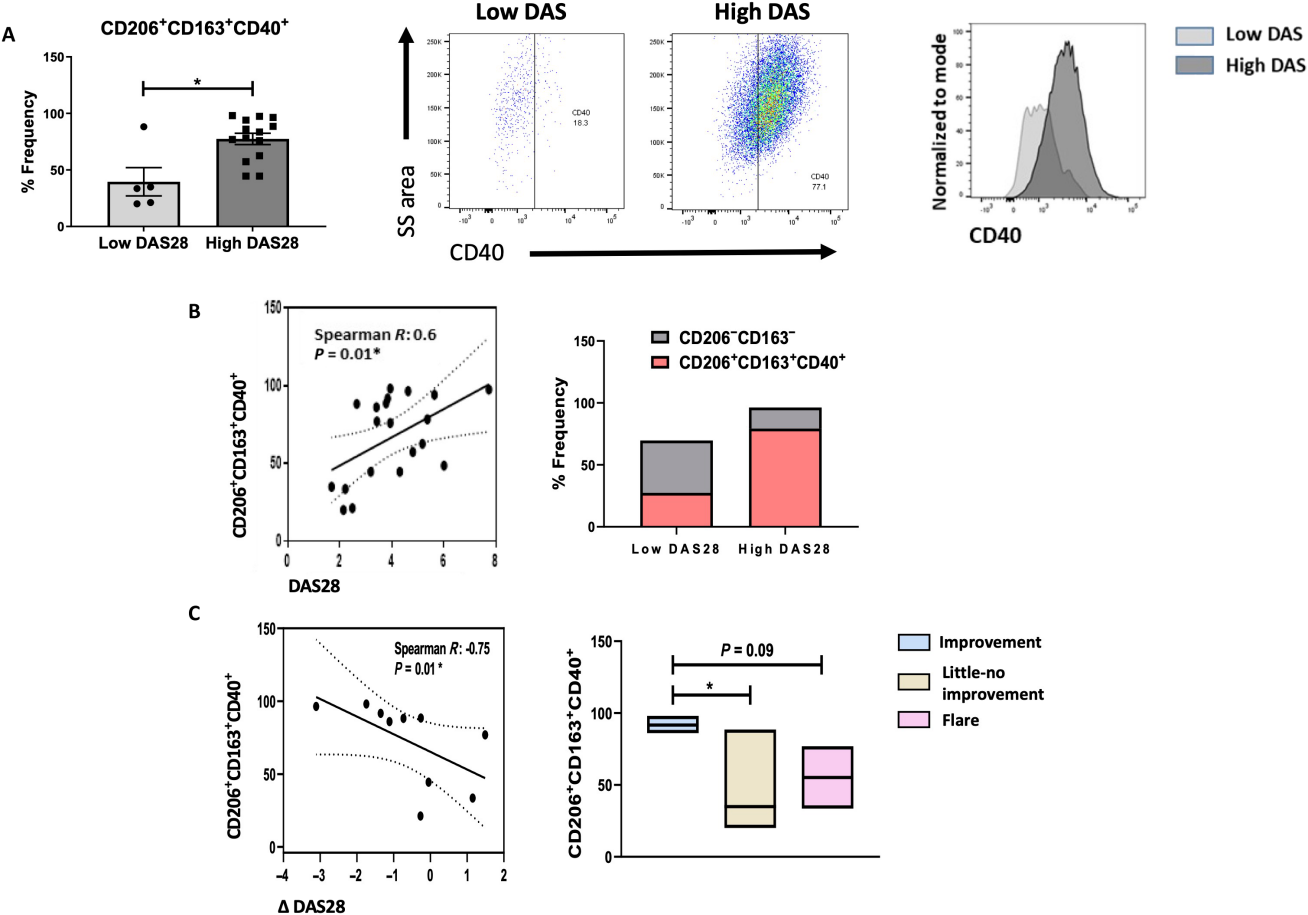
CD206^+^CD163^+^ RA macrophages correlate with RA disease activity and treatment response. Analysis of CD206^+^CD163^+^CD40^+^ based on RA disease activity scores (DAS28). Low DAS28 indicates less than 2.6 with high DAS28 greater than 2.6. (**A**) Dot plot, representative flow cytometry plots, and relative histogram of CD40 expression on double-positive CD206^+^CD163^+^ macrophage population in high versus low disease activity. (**B**) Correlation and relative proportion bar chart representing the relationship between baseline CD206^+^CD163^+^CD40^+^ expression and DAS28. (**C**) Correlation and bar charts representing the relationship between baseline CD206^+^CD163^+^CD40^+^ expression and change in DAS28 upon 1-year follow-up. Data are presented as mean ± SEM, using Mann-Whitney *U* test, *P < 0.05.

To investigate the clinical significance of the synovial tissue macrophage subset on RA disease clinical parameters, patient samples were stratified according to high (>2.6) versus low (<2.6) RA disease activity scores (DAS28). CD206^+^CD163^+^CD40^+^ macrophages are significantly increased in RA synovial tissue from patients with higher disease activity, indicated by percentage frequency dot plots, relative proportion, and CD40 histogram analysis (Fig. 2A, *P* < 0.05). In addition, the frequency of the CD206^+^CD163^+^CD40^+^ subset significantly correlates with DAS28 scores (Fig. 2B, *r* = 0.6, *P* < 0.01). Analysis of patients with RA with follow-up clinical data demonstrated that higher baseline expression of CD206^+^CD163^+^CD40^+^ predicted a greater response to treatment at 1-year follow-up compared to those with minimal response or flare (Fig. 2C). This is also evident in the inverse relationship between change in DAS28 and CD206^+^CD163^+^CD40^+^ baseline expression after 1-year follow-up (Fig. 2C, *r* = −0.75, *P* < 0.01). Together, these data indicate a key role of this dominant population in driving RA disease activity and severity, supporting their pathogenic role in RA with baseline levels predicting response to therapy.

### CD206^+^CD163^+^ RA synovial tissue macrophages are a pathogenic macrophage subset

**Fig. 3.**
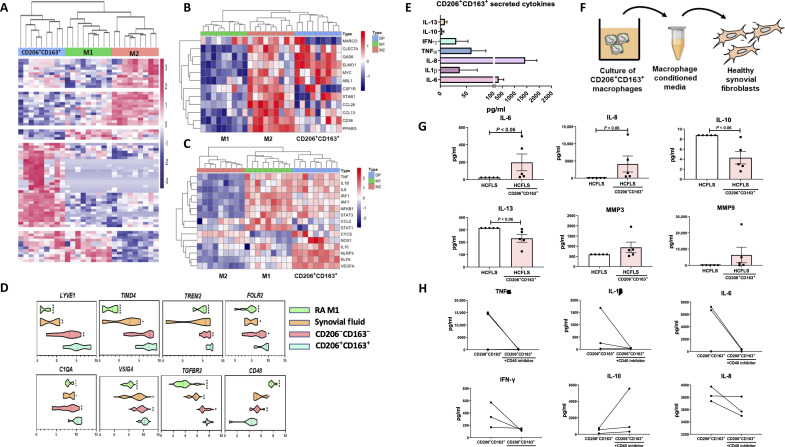
Pathogenic role of transitional CD40-expressing CD206^+^CD163^+^ macrophages. (**A**) Clustered heatmap displaying unsupervised hierarchical clustering of genes that are distinct in RA synovial tissue CD206^+^CD163^+^ macrophages (*n* = 9) and patient-matched polarized RA monocyte-derived M1 and M2 macrophages (*n* = 9). Heatmaps representing log-normalized expression values of (**B**) the top M2-like and (**C**) M1-like genes in RA M1 and M2 macrophages and CD206^+^CD163^+^ RA synovial tissue macrophages (*n* = 9). (**D**) Violin plots representing log-normalized expression values of macrophage tissue-resident–associated genes in the CD206^+^CD163^+^ macrophages (*n* = 9) compared to CD206^−^CD163^−^ macrophages (*n* = 9), synovial fluid CD206^+^CD163^+^ (*n* = 4), and polarized RA M1 macrophages (*n* = 9) with medians marked by black dots. Data expressed as mean ± SEM using one-way analysis of variance (ANOVA) with Tukey’s multiple comparisons test, *P < 0.05, **P < 0.01, ***P < 0.001, ****P < 0.0001 significantly different from each other. (**E**) Bar graphs represent cytokines secreted from sorted RA synovial tissue CD206^+^CD163^+^ macrophages measured by MSD assay. (**F**) Schematic of experimental workflow of CD206^+^CD163^+^ macrophage conditioned media on healthy synovial fibroblasts. (**G**) Dot plots of cytokine expression following the addition of CD206^+^CD163^+^ macrophage conditioned media on healthy synovial fibroblasts (*n* = 4). (**H**) Line plots indicating sorted CD206^+^CD163^+^ synovial tissue macrophage cytokine expression with or without preincubation with CD40 inhibitor (*n* = 3). Data are presented as mean ± SEM with each symbol representing a different sample. Statistical analysis using Mann–Whitney *U* test.

Despite being classified by two M2-like markers, CD206 and CD163, this macrophage population does not appear to be a typical M2 macrophage, most notably due to high coexpression of CD40. We therefore hypothesize that the enriched RA CD206^+^CD163^+^ macrophage subset is an intermediate or transitional subset of tissue macrophage, which does not adhere to the traditional dichotomous M1 versus M2 model, instead lying somewhere in between a wide spectrum of macrophage activation states. Unsupervised hierarchical clustering of the total gene expression dataset demonstrates that RA CD206^+^CD163^+^ macrophages cluster separately from RA M1 and M2 macrophages (Fig. 3A). The notable transcriptional differences are further reflected in volcano plots, whereby CD206^+^CD163^+^ synovial tissue macrophages display clear transcriptional differences compared to M1 and M2 (fig. S2B), with red data points indicating differentially expressed genes (DEGs) (adjusted *P* value < 0.05). This confirms our hypothesis that CD206^+^CD163^+^ macrophages enriched in the RA synovium are in a transitionary state and transcriptionally distinct from the polar dichotomy of pure monocyte-derived M1 and M2 macrophages.

Genes associated with M2 macrophages were enriched in CD206^+^CD163^+^ macrophages compared to RA M1 macrophages, suggesting that these cells are “M2-like” (Fig. 3B). However, examining inflammatory genes classically associated with the pro-inflammatory M1 macrophage, CD206^+^CD163^+^ macrophages also highly express these genes in comparison to pure RA M2 macrophages (Fig. 3C). Inflammatory genes such as *TNF, IL-1B, IL-6*, and *NFΚB* are all enriched in both RA M1 and CD206^+^CD163^+^ macrophages compared to M2 (Fig. 3C). CD206^+^CD163^+^ macrophages express *NLRP3, KLF6,* and *IL-10* and the pro-angiogenic factor *VEGFA* compared to both RA M1 and M2 (Fig. 3C). In addition, differential signal transducer and activator of transcription (STAT) expression is observed whereby *STAT3* is enriched in both RA M1 and CD206^+^CD163^+^ macrophages compared to M2 while *STAT1* is preferentially enriched in RA M1 only (Fig. 3C). Genes associated with phagocytosis are specifically enriched in CD206^+^CD163^+^ synovial tissue macrophages compared to M1 and in some cases compared to both M1 and M2 (*MARCO* and *ELMO*) (Fig. 3B). These data reinforce the observation that CD206^+^CD163^+^ macrophages residing in RA synovial tissue are phenotypically M2-like but have acquired strong pro-inflammatory “M1-like” properties upon initiation of active disease and thus display a transitionary phenotype.

To determine the origin of these macrophages, we examined tissue residency genes including *LYVE1, TIMD4, TREM2, FOLR2, C1QA, VSIG4*, and *TGFBR2*A. A significant increase in all “tissue-resident”–associated genes in synovial tissue CD206^+^CD163^+^ macrophages compared to double-negative CD206^−^CD163^−^ macrophages, RA synovial fluid CD206^+^CD163^+^ macrophages, and RA M1 macrophages was observed (Fig. 3D, *P* < 0.01; *P* < 0.05; *P* < 0.001 respectively). In contrast, *CD48*, a monocytic signature gene, is reciprocally significantly blunted in CD206^+^CD163^+^ synovial tissue macrophages compared to the three other populations (Fig. 3D, *P* < 0.01; *P* < 0.05; *P* < 0.001, respectively). Ingenuity pathway analysis (IPA) allowed for identification of key signaling pathways distinct to synovial tissue double-positive macrophages compared to synovial fluid (fig. S2C), suggesting that the phenotypic and functional differences observed between synovial tissue and fluid CD206^+^CD163^+^ may be due to differing ontogeny. Together, these data suggest that CD206^+^CD163^+^ synovial tissue macrophages are not derived from infiltrating immune cells from the peripheral circulation but are tissue resident.

Consistent with this, we cultured sorted RA synovial tissue CD206^+^CD163^+^ macrophages and demonstrated spontaneous secretion of key pro-inflammatory mediators IL-6, IL-8, IL-1β, and tumor necrosis factor–α (TNFα; Fig. 3E). Furthermore, culture of healthy synovial fibroblasts (HCFLS) with CD206^+^CD163^+^ conditioned media (Fig. 3F) greatly induced HCFLS secretion of IL-6 and IL-8 coupled with blunted expression of the anti-inflammatory IL-10 and IL-13 (Fig. 3G, *P* = 0.06). When we examine the effect of polarized monocyte-derived macrophage media on HCFLS, we note that CD206^+^CD163^+^ sorted macrophages display similar responses to M1-inflammatory macrophages with regard to IL-6 and IL-8 secretion, with synovial tissue macrophage conditioned media eliciting the greatest secretion of IL-8 from HCFLS (Fig. 3G and fig. S3A). In contrast, monocyte-derived macrophage conditioned media enhances IL-10 and IL-13 secretion from HCFLS, significantly so with M1-conditioned media, while CD206^+^CD163^+^ macrophages decrease expression of these anti-inflammatory mediators (fig. S3A, *P* < 0.05). Moreover, expression of matrix metalloproteinase 3 (MMP3) and MMP9 is up-regulated in HCFLS cultured with CD206^+^CD163^+^ conditioned media, suggesting induction of an invasive phenotype (Fig. 3G). Given the coexpression of CD40 on CD206^+^CD163^+^ macrophages, we next examined whether CD40 inhibition would reinstate the immunoregulatory phenotype of these macrophages. Treatment of RA sorted CD206^+^CD163^+^ macrophages with a CD40-TRAF6 signaling inhibitor resulted in decreased expression of TNFα, IL-1β, IL-6, and interferon-γ (IFN-γ) in two patients with a variable response observed for the third patient, indicative of patient heterogeneity (Fig. 3H). This decreased pro-inflammatory response was simultaneously observed with an increase in IL-10 production, a typically anti-inflammatory cytokine.

### CD40-expressing CD206^+^CD163^+^ synovial tissue macrophages are dominant in RA with disruption of CX_3_CR1 expression upon initiation of RA disease

**Fig. 4.**
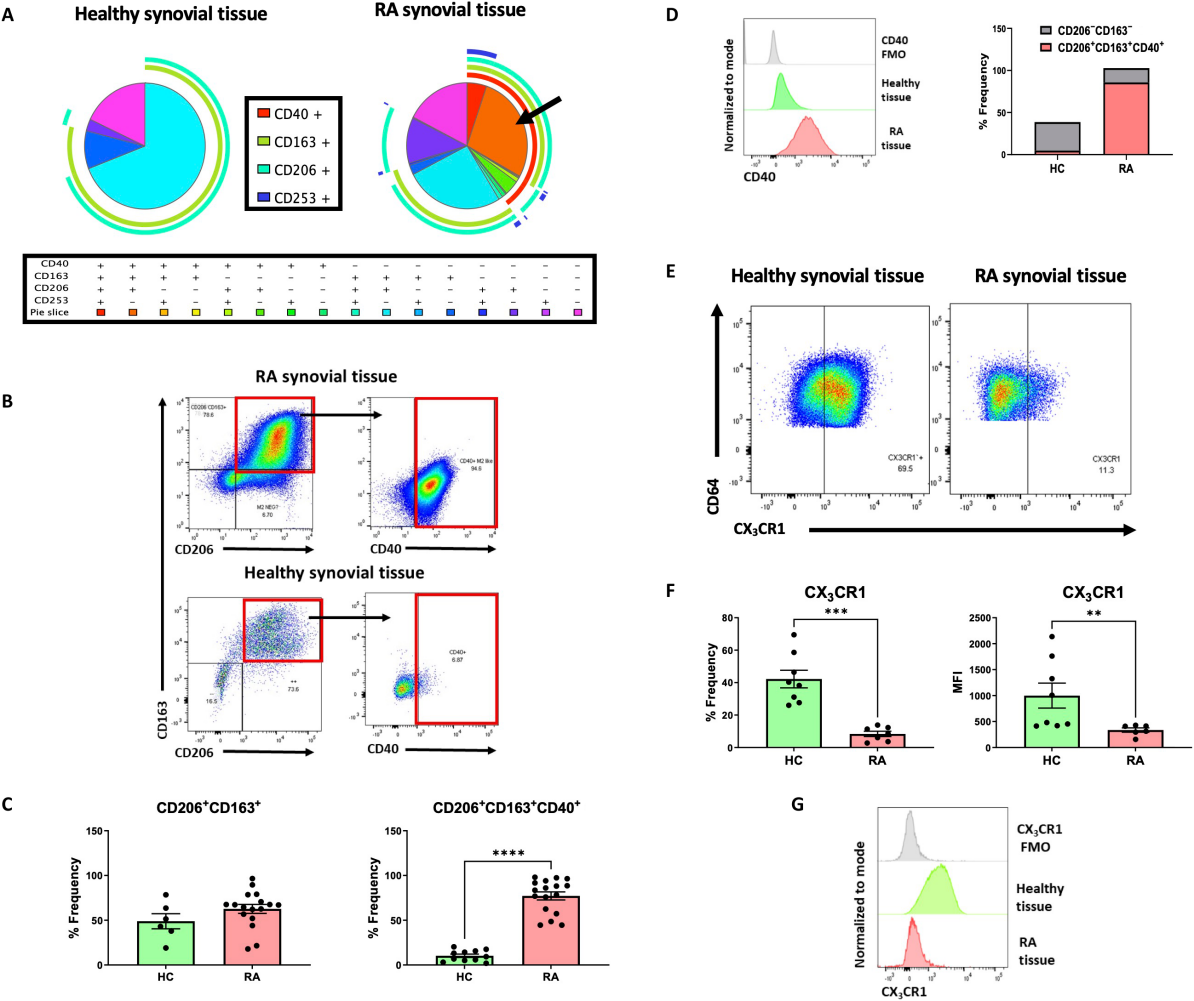
Synovial tissue macrophage expression in healthy versus RA synovial tissue. (**A**) SPICE analysis of macrophage subsets in an average of healthy synovial tissue (*n* = 3) and RA synovial tissue (*n* = 9). (**B**) Representative flow cytometry plots indicating the expression of double-positive CD206^+^CD163^+^ and coexpression of CD40 macrophages in healthy and RA synovial tissue. (**C**) Dot plots indicating the percentage frequency of CD206^+^CD163^+^ and CD206^+^CD163^+^CD40^+^ macrophage subsets in RA synovial tissue (*n* = 17) compared to healthy control tissue (*n* = 6 to 11) along with (**D**) representative histogram and relative proportion graphs of CD40 expression on the double-positive CD206^+^CD163^+^ macrophage subset in healthy versus RA synovial tissue. (**E**) Representative flow cytometry dot plots of CX_3_CR1 expression with accompanying (**F**) quantification of percentage frequency and MFI and (**G**) representative histogram of CX_3_CR1 expression in healthy (*n* = 8) and RA (*n* = 7) synovial tissue macrophages. Data are presented as mean ± SEM with each symbol representing a different sample. Statistical analysis using Mann-Whitney *U* test, **P < 0.01, ***P < 0.001, ****P < 0.0001 significantly different from each other.

We next investigated whether this enriched CD40-expressing synovial macrophage population is present in the healthy synovium. SPICE analysis demonstrated a marked increase in the diversity of macrophage subsets in RA diseased tissue compared to healthy synovium (Fig. 4A). Specifically, the dominant population of macrophages residing in healthy synovium are the typically homeostatic CD206^+^CD163^+^ subtype as indicated by the light blue segment of the pie chart (Fig. 4A), with comparable expression compared to the RA synovium. However, coexpression of CD40 on CD206^+^CD163^+^ macrophages is completely absent in healthy synovial tissue, indicated by representative flow plots and accompanying quantification (Fig. 4, B to D, *P* < 0.05). Hence, we propose that the double-positive CD206^+^CD163^+^ macrophage subset dominates the synovium in the steady state, yet upon initiation of active RA disease, this subset transforms to become an activated transitionary subset of macrophages capable of expressing high levels of CD40.

We also examined whether the protective barrier-like CX_3_CR1-expressing macrophages are present in the healthy joint as previously described in a mouse model of arthritis by Culemann *et al.* (*29*). In line with the previously reported mouse data, we report in humans a significant reduction in these protective CX_3_CR1-expressing macrophages in RA compared to the healthy controls (Fig. 4, E to G, *P* < 0.05). Together, these data indicate a dominance of disease-specific enhanced CD40 expression coupled with blunted CX_3_CR1 expression in macrophages residing within the inflamed RA synovium.

In addition, to relatively compare our data to the previously published study by Alivernini *et al.* (*30*), we examined CD40 expression on MerTK^+^CD206^+^ synovial tissue macrophages and demonstrate that the high level of CD40 coexpression observed on CD206^+^CD163^+^ RA macrophages in our study is also present on MerTK^+^CD206^+^ synovial tissue macrophages in RA (fig. S3, C and D). Therefore, the data presented in this manuscript describing CD206^+^CD163^+^ macrophages in health and disease is distinct from Alivernini *et al.* but both studies are crucial to extending our understanding on the critical role of macrophages in driving synovial inflammation.

### Single-cell transcriptomic analysis of synovial tissue macrophages from health to disease

**Fig. 5.**
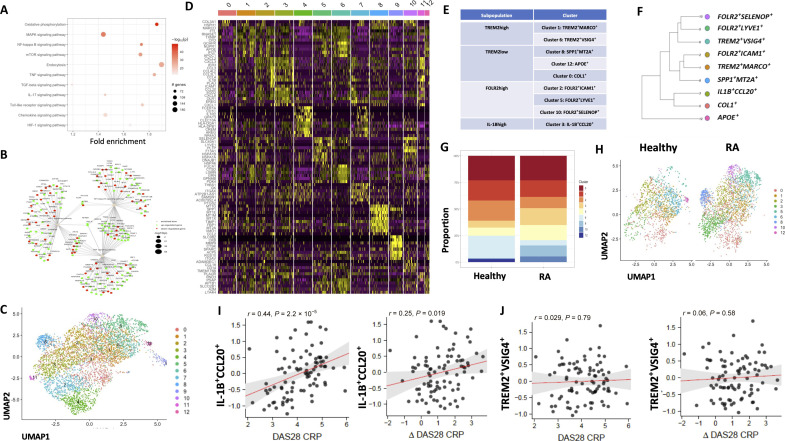
scRNA-seq defines synovial tissue macrophage heterogeneity from health to disease. (**A**) Analysis of pathways enriched in CD206^+^CD163^+^ macrophages in healthy (*n* = 4) and RA (*n* = 4) synovial tissue, color intensity represents significance, and dot size denotes the number of genes within each pathway that are differentially expressed. (**B**) Term plot of the indicated pathways with significant enrichment in RA compared with healthy control macrophages. Color indicates up- or down-regulation of specific genes within the pathway and dot size represents statistical significance of change. (**C**) UMAP representation of 12 synovial tissue myeloid clusters based on 67,908 synovial tissue cells identified by high-dimensionality scRNA-seq analysis. (**D**) Heatmap of the top 20 DEGs per cluster. (**E**) Proposed classification and (**F**) hierarchical clustering of human synovial tissue macrophages. (**G**) Proportional cluster abundance between patients with established RA and healthy control individuals. (**H**) UMAP depicting synovial tissue macrophage cluster distribution between RA and healthy synovium. Two-tailed Spearman’s correlation between synovial expression of (**I**) IL-1B^+^CCL20^+^ and (**J**) SPP1^+^MT2A^+^ synovial tissue macrophage clusters with DAS28 CRP and changes in DAS28 CRP in the PEAC cohort (*n* = 90).

Next, we performed single-cell transcriptomic profiling of synovial tissue cells from patients with RA and healthy individuals to provide a unique myeloid atlas from health to disease. Clinical information of patients at the time of arthroscopy and number of cells sequenced per donor are summarized in Tables 4 and 5, respectively, with 5417 synovial tissue myeloid cells analyzed in total. Given the enrichment of CD206^+^CD163^+^ macrophages in both healthy and RA synovial tissue, we initially examined this signature using single-cell transcriptomic analysis. CD206^+^CD163^+^ macrophages residing within the inflamed RA synovium express inflammatory genes such as *SPP1* (osteopontin) and *SOD2* (a superoxide anion overproduced in joint inflammation) (*42*), and healthy synovial tissue macrophages, in contrast, express markers such as *TREM2* and *APOE*, thought to play an important role in immunoregulation (*43*) (fig. S4A). Pathway enrichment identified several pathogenic pathways enriched in RA synovial tissue macrophages. These pathways include TNF signaling pathways, NF-κB signaling pathway, and HIF-1α signaling pathways (Fig. 5A). Common genes and the up-regulation or down-regulation of specific members of these pathways in RA synovial tissue macrophages are depicted in Fig. 5B. In addition, CD206^+^CD163^+^ macrophages display an enriched tissue-resident gene signature compared to CD206^−^CD163^−^ macrophages (similarly observed in Fig. 3D), an effect enhanced in healthy control synovial tissue macrophages when compared to RA (fig. S4B). Moreover, we note that the NF-κB signaling module is specifically enriched in RA CD206^+^CD163^+^ macrophages when compared to healthy and CD206^−^CD163^−^ synovial tissue macrophages (fig. S4C). This is particularly interesting as not only is NF-κB a hallmark of chronic inflammatory diseases, playing a key role in mediating innate pro-inflammatory mechanisms (*44*), but CD40 plays a key role in its activation. Healthy control synovial tissue macrophages also display enhanced expression of gene modules associated with oxidative phosphorylation compared with RA with similar glycolysis expression levels based on Kyoto Encyclopedia of Genes and Genomes (KEGG) oxidative phosphorylation and glycolytic pathway analysis, respectively (fig. S4, D and E).

**Table 4.** Patient demographics of RA synovial tissue samples used for scRNA-seq analysis. Clinical information of patients with RA for synovial tissue samples included in this study (*n* = 4). RF, ACPA, CRP, erythrocyte sedimentation rate (ESR), swollen 28-joint count (SJC28), tender 28-joint count (TJC28), DAS28, disease duration, and mediations at the time of arthroscopy are shown.

Characteristics	RA (*n* = 4)
Number (M/F)	4 (1/3)
Age mean, range	57.5 (39–83)
APCA, pos/neg	3/1
RF, pos/neg	3/1
CRP, mean ± SD	14.5 ± 8.3
ESR, mean ± SD	45.6 ± 27.7
SJC28, mean ± SD	5.4 ± 7
TJC28, mean ± SD	7.8 ± 9
DAS28, mean ± SD	4.6 ± 1.1
Disease duration, mean ± SD	4.5 ± 8.3
Medications	Naïve

**Table 5.** Number of cells sequenced per donor used for scRNA-seq analysis.

Disease	Patient	No. of cells sequenced
Healthy	HC002	2338
	HC004	18,882
	HCBX003	3113
	HCBXN001	21,104
	HCBXN005	5279
RA	RA1	10,905
	RA2	4666
	RA4	14,509
	RA5	12,724

Next, using nonlinear, stochastic UMAP analysis, 13 subclusters of synovial tissue myeloid cells were identified (Fig. 5C). Four of these clusters were identified as dendritic cell subclusters and thus excluded from further analysis, leaving nine distinct macrophage subclusters. These distinct synovial tissue macrophage clusters were further characterized using gene expression analysis of the top 20 DEGs per cluster and hierarchical clustering analysis (Fig. 5D and fig. S5A). Following these approaches, subclusters were further classified into four subpopulations identified as TREM2high, TREM2low, FOLR2high, and IL-1Bhigh with proposed taxonomy demonstrated in Fig. 5 (E and F). The TREM2high population contains two subclusters, TREM2^+^MARCO^+^ and TREM2^+^VSIG4^+^; these two clusters display homeostatic phagocytic macrophage gene signatures including the scavenger receptor *TIMD4* and the lipid-binding protein *APOE*. These clusters also have minimal inflammatory gene expression as depicted in Fig. 5D and display similar gene expression profiles to the TREM2high synovial tissue macrophage subpopulation described by Alivernini *et al.* (*30*). The TREM2low population comprises three macrophage clusters: SPP1^+^MT2A^+^, APOE^+^, and COL^+^. The SPP1^+^MT2A^+^ cluster is an inflammatory macrophage cluster characterized by the abundance of inflammation-triggering alarmins *S100A8* and *S100A9* and *SPP1*. Expression of SPP1 and S100A9 positively correlate with RA disease activity (*30*). The FOLR2high subpopulation consists of three clusters, FOLR2^+^ICAM^+^, FOLR2^+^LYVE1^+^, and FOLR2^+^SELENOP^+^. The latter two clusters display very similar homeostatic gene signatures with coexpression of *LYVE1, FI3A1, STAB1*, and *SELENOP*. In contrast, the FOLR2^+^ICAM^+^ cluster displays a mixed gene signature with high expression of many inflammatory markers such as *IL-1B* and *CLL4*. Last, a pro-inflammatory IL-1Bhigh macrophage cluster was also identified defined with coexpression of *CCL20* (Fig. 5, D to F).

Differential abundance of synovial macrophage clusters is observed in healthy control individuals and those with active RA disease (Fig. 5, G and H). The two clusters identified as pro-inflammatory macrophage populations (IL-1B^+^CCL20^+^ and SPP1^+^MT2A^+^) are specifically enriched in RA compared to healthy synovial tissue; in fact, the SPP1^+^ cluster appears to be absent in healthy synovium, indicating that it is a disease-specific myeloid subpopulation (Fig. 5, G and H). The proportion of COL^+^ macrophages, characterized by enriched gene expression of *COL3A1*, which encodes for type III collagens, are similar in healthy and RA synovial tissues (Fig. 5, G and H). FOLR2^+^ICAM1^+^ macrophages are proportionally enriched in healthy synovial tissue compared to RA and are characterized by many pro-inflammatory cytokines and chemokines such as *CCL4, TNF,* and *IL-1B*, indicating that they may form the first line of innate defense against pathogens in the synovial joint. The TREM2high subpopulation comprises two homeostatic macrophage clusters identified as MARCO^+^ and VSIG4^+^ macrophages, specifically enriched in healthy synovium compared to active disease (Fig. 5, G and H). TREM2^+^VSIG4^+^ macrophages are scarce in RA synovial tissue yet make up the highest proportion of healthy synovial tissue macrophages while IL-1B^+^CCL20^+^ macrophages comprise the largest proportion of macrophages residing within the inflamed RA synovium. Given the specific cluster abundance in RA and healthy synovium, we next analyzed the transcriptomic profile of these divergent macrophage subpopulations. Pathway enrichment analysis indicates oxidative phosphorylation and endocytosis as key signaling pathways in the TREM2highVSIG4^+^ macrophage population while the inflammatory IL-1B^+^CCL20^+^ macrophages enriched in RA synovial tissue display enrichment of signaling pathways such as cell adhesion, antigen processing, and presentation in addition to key inflammatory signaling pathways (NF-κB and PI3K signaling; fig. S5, B and C).

Similar to what we had earlier observed in CD206^+^CD163^+^ synovial tissue macrophages in Fig. 1 (F and G) and fig. S4 (D and E), healthy control synovial tissue macrophage clusters display enhanced expression of oxidative phosphorylation gene modules when compared with RA (fig. S6A). Specifically, SPP1^+^MT2A^+^ macrophages display the highest expression gene modules associated with glycolysis when compared to all other synovial tissue macrophage clusters, an effect that is markedly enhanced in SPP1^+^MT2A^+^ macrophages residing within the inflamed RA synovium compared to healthy synovium (fig. S6B). In contrast, homeostatic TREM2^+^ macrophage clusters display enhanced oxidative phosphorylation gene modules, particularly in those residing in healthy synovium (fig. S6A).

To further examine the clinical relevance, we investigated these macrophage signatures in a large cohort of early treatment-naïve patients, using the Pathobiology of Early Arthritis Cohort (PEAC) (*45*). Analysis confirms that the IL-1B^+^CCL20^+^ macrophage cluster is enriched in patients with RA and positively correlates with RA disease activity (*r* = 0.44, *P* = 2.2 × 10^−5^) and change in DAS28CRP between baseline and 6 months following methotrexate-based therapy (*r* = 0.25, *P* = 0.019) (Fig. 5I). In addition, most IL-1B^+^CCL20^+^ cluster genes significantly correlate with clinical parameters such as RF, CRP, ESR, DAS28, and VAS (fig. S7A). In contrast, TREM2^+^VSIG4^+^, macrophage clusters that dominate healthy synovium, were not associated with clinical parameters (Fig. 5J and fig. S7B). Thus, together, these data indicate a key role of the IL-1B^+^CCL20^+^ inflammatory macrophage subset in disease pathogenesis, which importantly appears to be an early activation state.

Moreover, previous studies have also demonstrated the presence of three distinct synovial pathotypes based on cellular and molecular analysis of synovial tissue: lymphoid (dominated by the presence of B cells and myeloid cells), myeloid (myeloid rich, B cell poor), and fibroid characterized by the strong presence of stromal cells (*45*, *46*). We therefore examined the IL-1B^+^CCL20^+^ and TREM2^+^VSIG4^+^ macrophage clusters with respect to their correlations to synovial pathotypes. Both macrophage clusters are associated with lymphoid and myeloid pathotypes compared to fibroid, and TREM2^+^VSIG4^+^ macrophages display greater enrichment in the myeloid pathotype, compared to the inflammatory IL-1B^+^CCL20^+^ subset (fig. S8, A and B).

### Myeloid cellular interactions in the inflamed RA synovium

**Fig. 6.**
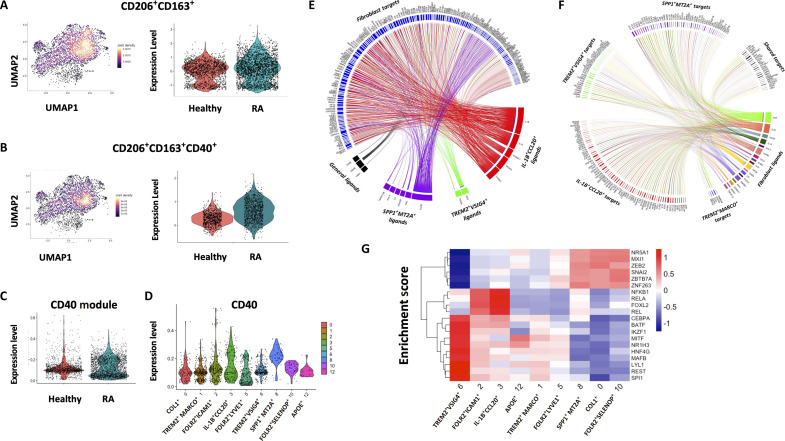
Synovial tissue macrophage CD40 expression and cellular interactions. (**A**) UMAP illustrating CD206^+^CD163^+^ mRNA expression in synovial tissue macrophages. Violin plot representing the expression level of CD206^+^CD163^+^ in healthy and RA synovium. (**B**) UMAP illustrating CD206^+^CD163^+^CD40^+^ mRNA expression in synovial tissue macrophages. Violin plot representing the expression level of CD206^+^CD163^+^CD40^+^ in healthy and RA synovium. (**C**) Violin plot demonstrating CD40 signaling module expression between healthy and RA synovium. (**D**) Violin plot representing CD40 gene expression levels across all synovial tissue macrophage clusters. (**E** and **F**) Circos plots depicting the top predicted receptor and ligand interactions between specific indicated macrophage clusters and synovial tissue fibroblasts. (**G**) DoRothEA analysis of transcription factor usage by RA compared with healthy control synovial tissue macrophage clusters, based on expression of known downstream ligands.

scRNA-seq analysis also demonstrated that the double-positive CD206^+^CD163^+^ macrophage signature is dominant in the synovium with similar frequencies observed in both healthy and RA synovial tissue (Fig. 6A), while the CD206^+^CD163^+^ coexpressing CD40 triple-positive signature and CD40 signaling module is uniquely enriched in RA synovial tissue compared to HC (Fig. 6, B and C), thus confirming our flow cytometric analysis (Fig. 4). When we examine CD40 expression across the nine identified synovial tissue macrophage clusters, IL-1B^+^CCL20^+^ and SPP1^+^MT2A^+^ macrophage clusters, both uniquely enriched in RA synovial tissue compared to healthy tissue, display the highest expression of CD40 in synovial tissue macrophages (Fig. 6D). This further highlights the importance of CD40 as a synovial tissue macrophage activation marker in RA. CD40 expression across all myeloid subclusters, including dendritic cell clusters, can also be observed in fig. S9B.

As scRNA-seq analysis was performed on unsorted synovial tissue single-cell suspensions, this allowed us to examine potential networks of myeloid cell interactions, reflective of the joint microenvironment. Synovial fibroblasts are key resident cells of the synovium and, thus, myeloid-stromal cell cross-talk was investigated. Specifically, we investigated the cross-talk between stromal cells and four key macrophage clusters, namely, the TREM2 homeostatic clusters (TREM2^+^MARCO^+^ and TREM2^+^VSIG4^+^) and the opposing inflammatory clusters (IL-1B^+^CCL20^+^ and SPP1^+^MT2A^+^). We demonstrate that IL-1B^+^CCL20^+^ cluster ligands drive an invasive phenotype in synovial fibroblasts with induction of many MMPs (Fig. 6E), similar to the effects observed in Fig. 4G. In addition, SPP1^+^MT2A^+^ macrophage cluster ligands drive the expression of type I, IV, and V collagen in synovial fibroblasts (Fig. 6E). Examining the effect of synovial fibroblast ligands on inflammatory macrophage cluster receptors, we demonstrated that fibroblast-derived ligands drive expression of many chemokines (*CLL2* and *CCL5*) and MMPs in the SPP1^+^MT2A^+^ synovial tissue macrophage cluster and also induce *CD40* expression in this macrophage subpopulation (Fig. 6F). In addition, we also conducted analysis on myeloid–T cell cross-talk in the synovium. Here, we demonstrate that synovial tissue macrophage clusters induce many T cell activation markers such as *CD44*, *CD69*, and *CD226* along with many chemokine and chemokine receptors (*CCL3*, *CCL4*, *CXCR4*, and *CXCL8*) (fig. S10A). Moreover, T cell–derived ligands induce inflammatory gene expression in synovial tissue macrophages: *IL-1B*, *IL-6*, and *TNF* in the IL-1B^+^CCL20^+^ subcluster and *CD40* in SPP1^+^MT2A^+^ macrophages (fig. S10B).

Next, to identify regulators of the transcriptional profiles of distinct synovial tissue subsets, we performed transcription factor usage estimation by analyzing the expression of known, transcription factor-regulated genes that are differentially expressed across macrophage subsets. The two inflammatory clusters, IL-1B^+^CCL20^+^ and SPP1^+^MT2A^+^, and the moderately inflammatory FOLR2^+^ICAM1^+^ macrophage cluster all display enhanced activity of NF-κB–like transcription factors (NF-κB1, REL, and RELA) (Fig. 6G).

### Pathogenic synovial tissue macrophage signature predates clinical manifestations of disease

**Fig. 7.**
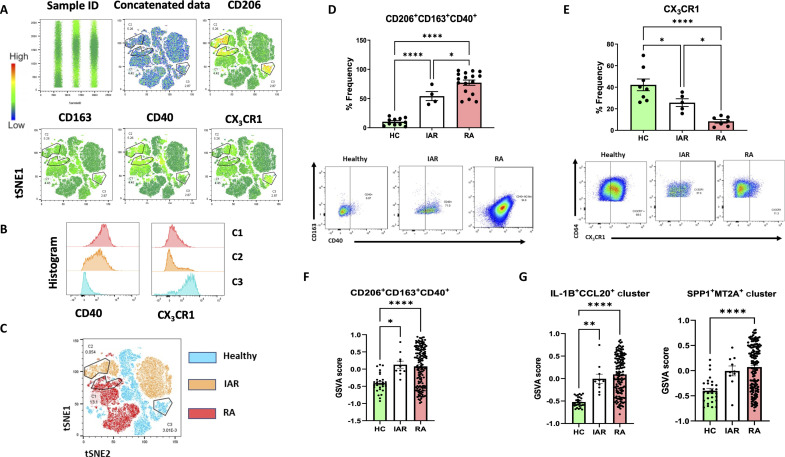
Synovial tissue macrophage expression in individuals at risk of developing rheumatoid arthritis. (**A**) Multiparametric flow cytometric data of total CD64^+^ macrophages subjected to tSNE algorithm following data concatenation of one healthy individual at risk and RA synovial tissue. Data concatenation and relative expression for the indicated parameters are shown. (**B**) Identification of three populations of CD206, distinguished by CD40 and CX_3_CR1 expression, and (**C**) expression of three clusters in healthy, IAR, and RA synovial tissue. Representative flow cytometry dot plots and accompanying percentage frequency quantification of (**D**) CD206^+^CD163^+^CD40^+^ in healthy (*n* = 11), arthralgia (*n* = 4), and RA (*n* = 17) and (**E**) CX_3_CR1 in healthy (*n* = 8), arthralgia (*n* = 5), and RA (*n* = 6) synovial tissue macrophages. (**F**) Dot plots of expression of CD206^+^CD163^+^CD40^+^ and (**G**) IL-1B^+^CCL20^+^ and SPP1^+^ MT2A^+^ gene signatures in IAR (*n* = 10) and established RA (RA) (*n* = 85) compared to normal healthy synovium (*n* = 44) synovial tissue. Data expressed as mean ± SEM using one-way ANOVA with Tukey’s multiple comparisons test, *P < 0.05, **P < 0.01, ****P < 0.0001 significantly different from each other.

Last, we investigated how early in disease this pathogenic macrophage signature is present. It is well documented that circulating autoantibodies and inflammatory markers can be present long before clinical signs and symptoms of arthritis (*40*, *47*–*49*). To assess whether the loss of macrophage homeostasis identified in established RA synovium is an event that occurs before disease onset, synovial tissue macrophages from IAR of RA were examined. Initially, we performed unbiased, unsupervised, multidimensional analysis of flow cytometric data on healthy, IAR, and established RA synovial samples using the t-distributed stochastic neighbor embedding (tSNE) algorithm following pregating on CD64^+^ cells (*50*). Total CD64 cells were subjected to tSNE algorithm following data concatenation (Fig. 7A). Analysis identified three “clusters” of macrophages based on CD206 expression (Fig. 7A). Further analysis demonstrated that “C1” and “C2” also express CD40 while “C3” highly expresses CX_3_CR1 (Fig. 7B). Healthy (blue), IAR (orange), and RA (red) synovial tissue macrophages can be distinguished based on these clusters with the two CD40-expressing CD206 “C1/2” observed in IAR and RA synovial tissue and with the CX_3_CR1-expressing CD206 macrophage C3 unique to healthy synovium (Fig. 7C).

To investigate this further, we validated this in a larger cohort, and notably, analysis of synovial tissue from IAR of developing RA revealed a significant stepwise increase of CD40-expressing CD206^+^CD163^+^ macrophages from healthy to IAR to RA (Fig. 7D, *P* < 0.05). In parallel, a stepwise significant decrease in CX_3_CR1^+^ macrophages from RA to IAR to healthy was also observed (Fig. 7E, *P* < 0.05). These data suggest in human disease that disruption of macrophage homeostasis (protective barrier breakdown) coupled with acquisition of pathogenic CD40-driven phenotype occurs very early in RA disease pathogenesis, indeed before clinical manifestations of disease.

Last, bulk RNA-seq analysis of synovial tissue from healthy donors, IAR, and patients with established RA was performed on a previously described dataset (*51*). Gene set enrichment analysis reveals an early enrichment of the CD206^+^CD163^+^CD40^+^ signature in the synovium of at-risk individuals, before the onset of disease (*P* < 0.05) compared to healthy tissue (Fig. 7F). In addition, we examined the signature of the two inflammatory macrophage clusters identified by scRNA-seq in this bulk RNA-seq of synovial tissue from health to disease. Here, we note a significant enrichment in both IL-1B^+^CCL20^+^ and SPP1^+^MT2A^+^ macrophage clusters across the evolution of RA disease from healthy individuals to IAR and established RA (Fig. 7G, *P* < 0.05). Thus, studying these IAR may provide important clues in understanding the evolution of RA, and in identifying altered immune responses that may predict disease onset.

## DISCUSSION

Synovial tissue macrophages are critical orchestrators in the pathogenesis of RA. Historically recognized as amplifiers of inflammation and the main producers of TNF among other key pro-inflammatory cytokines, recent studies have facilitated a better understanding of the diverse roles these cells play in both health and disease. Synovial tissue macrophages comprise a multitude of macrophage subpopulations with individual functions in the synovial joint, capable of both shaping and being shaped by the synovial microenvironment. Here, we investigate the phenotypic, transcriptional, metabolic, and functional role of human synovial tissue macrophages from health to disease. We identify a CD40-expressing CD206^+^CD163^+^ macrophage population residing in the inflamed RA synovium, present before clinical signs and symptoms of RA pathogenesis that is significantly associated RA disease activity and treatment response. These macrophages are transcriptionally distinct with unique tissue-resident gene signatures and metabolic capacities and can activate healthy synovial fibroblast responses. Furthermore, single-cell transcriptomic profiling of synovial tissue cells from RA and HCs enabled the identification of nine distinct macrophage clusters with unique transcriptional profiles. TREM2high macrophages dominate the synovium in the steady state. However, upon initiation of active RA disease, IL-1B^+^CCL20^+^ and SPP1^+^MT2A^+^ macrophages become the principal synovial tissue macrophage subsets. These macrophages display heightened *CD40* gene expression, correlate with clinical parameters of disease, and are enriched before disease onset indicating this is an early disease signature. Investigations into innate immune signatures before disease such as this study may aid in the prediction of clinical responses and a better understanding of the evolution of disease from health to established RA.

The presence of a dominant macrophage subset in the RA synovium expressing high levels of CD206 and CD163 was initially unexpected considering the inflamed microenvironment these cells reside in. Are these macrophages attempting to resolve or promote inflammation? Or have these cells become dysfunctional, and thus fail to constrain the inflammatory response similar to RA regulatory T cells (*52*)? Here, we demonstrate that CD206^+^CD163^+^ macrophages also display strong coexpression of CD40, a costimulatory activation marker that contributes to pathogenic mechanisms via the CD40/CD40L pathway to sustain chronic inflammation in RA (*53*). CD40 ligand expression correlates with higher disease activity, with CD40 alleles known to be associated with risk of developing RA (*51*, *54*). Therefore, we propose that the inflammatory microenvironment of the RA joint acts as an “on demand” signal to transform CD206^+^CD163^+^ tissue macrophages from a homeostatic M2-like macrophage to a dysfunctional activated state. This is consistent with previous studies demonstrating that the inflamed synovial microenvironment can shape the phenotype and function of monocyte-derived macrophages (*55*–*59*).

Notable differences in the frequency and composition of macrophage subsets residing in RA synovial fluid and synovial tissue were also observed, highlighting microenvironmental and site-specific functions of synovial macrophages. While studies have examined levels of macrophage-derived cytokines in synovial fluids or synovial tissue in different arthropathies (*60*), no direct comparisons of RA synovial tissue and fluid macrophages have been explored. Therefore, this study suggests that analysis of RA synovial fluid does not clearly reflect the pathophysiological state of the RA synovium.

We also demonstrate that synovial tissue macrophages display gene signatures similar to, and distinct from, both RA M1-like and M2-like macrophages, indicating that they are an intermediate macrophage subset and that the M1/M2 dichotomous system may be an oversimplification of a more complex reality in vivo. Considering the marked difference in activation status and transcriptional variance observed in CD206^+^CD163^+^ macrophage subset residing in the synovial tissue compared to synovial fluid, and the presence of these cells in the healthy synovium, it is tempting to speculate that they are in fact tissue-resident macrophages. CD163^+^ macrophages are thought to identify macrophages at an advanced maturation stage (*61*, *62*), while CD163^+^ macrophages are unaffected by TNFi treatment (*62*); this persistence may be a further indication of tissue residency (*63*). Elucidating the exact origins of RA synovial tissue macrophages may provide opportunities to preferentially manipulate macrophages from a specific origin.

Considering the crucial role that metabolic reprogramming plays in shaping in vitro monocyte and macrophage polarization (*40*, *64*), the metabolic demands and the extent to which synovial tissue macrophages are metabolically plastic remain to be elucidated. Here, we assessed the bioenergetic status of RA synovial tissue macrophages in real time whereby CD206^+^CD163^+^ synovial tissue macrophages use oxidative phosphorylation as their main source of energy. This result was initially unexpected given the inflammatory phenotype of these cells and the well-documented links between glycolytic mechanisms and inflammatory function (*25*, *40*, *65*, *66*). However, the metabolic signature of synovial tissue macrophages may also shed light on their origin. It is thought that the infiltrating inflammatory macrophages are short-lived, terminally differentiated cells dependent on glycolysis for rapid immune activation while this short-term metabolic fix would possibly not support the in vivo functions and self-renewal capacities of tissue-resident macrophages, most likely requiring a bioenergetically “healthy” metabolic profile that would likely explain the reliance on mitochondrial respiration of this macrophage subset despite their inflammatory status (*67*, *68*).

Moreover, we identify a pathogenic role for CD206^+^CD163^+^ macrophages in the RA synovium, indicating that they spontaneously secrete many pro-inflammatory mediators, a phenotype that can be reversed upon inhibition of CD40-TRAF6 signaling. Complete inhibition of the CD40-CD40 ligand dyad has been associated with thromboembolic toxicity (*69*); thus, targeting downstream CD40 signaling such as CD40-TRAF6 interactions may be more useful (*70*). In contrast, in the setting of cancer, a recent study has developed a bispecific FAP-CD40 antibody, to induce CD40 stimulation solely in the presence of a fibroblast activation protein (FAP), to induce antitumor immunity (*71*). This study also demonstrates that the FAP-CD40 effect abrogates the systemic toxicity associated with nontargeted CD40 agonists (*71*). Effective blockade of the CD40/CD40L pathway results in the reduction of DAS28 in patients with RA (*72*). Thus, disruption of CD40-TRAF6 signaling in RA synovial tissue macrophages may be of therapeutic benefit.

Although synovial tissue macrophages have been identified as the most common immune cell present in the normal synovium (*73*, *74*), the function of macrophages in the healthy synovial tissue is poorly described. Here, we observe a marked increase in the diversity of macrophage subsets residing in RA compared to healthy synovial tissue biopsies with a dominance of CD206^+^CD163^+^ macrophages observed in both. This subset in healthy synovium lacks expression of the inflammatory marker CD40, which was expressed on most RA CD206^+^CD163^+^ macrophages. This is in line with reports of increased CD40 ligand expression in RA synovial tissue compared to healthy synovial biopsies with subsequent increases in CD40L-responsive genes (*51*). Notably, CX_3_CR1-expressing macrophages were almost absent in patients with active RA and indeed diminished before disease onset whereas they comprised approximately 45% of healthy synovial tissue macrophages. This is consistent with recent animal studies examining the protective role of CX_3_CR1 expressing macrophages in shielding the joint from inflammation. In collagen-induced and serum transfer arthritis mouse models, this anti-inflammatory macrophage barrier becomes disrupted upon induction of arthritis (*29*, *75*). Here, we demonstrate in the human synovium a stepwise decrease in the protective barrier CX_3_CR1^+^ macrophages from healthy individuals to IAR to patients with established RA, suggesting that this barrier breakdown commences at an early stage of disease and is progressive.

At a single-cell transcriptomic level, we demonstrate that the synovial tissue myeloid landscape is characterized by nine distinct macrophage subclusters. The predominance of TREM2high macrophages in healthy synovium is not wholly surprising. Studies have demonstrated that TREM2 acts to promote phagocytosis while simultaneously antagonizing myeloid responsiveness to inflammatory stimuli and, thus, acts to shut down immune activation (*76*–*78*). Alivernini *et al.* (*30*) describe similar resolvin-rich TREM2^+^ macrophage populations in HC synovial tissue. TREM2high populations described in this study are characterized by abundance of *APOE*, a lipid-binding protein. The small APOE^+^ macrophage cluster present in healthy synovium is not observed in RA synovial tissue. Recent studies have demonstrated that TREM2^+^ macrophages exert immunoregulatory properties by diverting cholesterol to excess lipid droplets via APOE (*79*–*81*). Therefore, the paucity of TREM2^+^ macrophage clusters in RA, and thus loss of their subsequent immunoregulatory actions, may result in unrestrained myeloid activation. In contrast, IL-1B^+^CCL20^+^ and SPP1^+^MT2A^+^ macrophages are abundant in RA synovial tissue yet scarce in healthy synovium. SPP1^+^ macrophages are hallmarked by enhanced gene expression of *SPP1* (osteopontin), which is an extracellular matrix glycoprotein that plays a critical role in bone remodeling and is also recognized as an inflammatory cytokine (*82*). Clinically, high levels of osteopontin have been detected in the synovium, plasma, and serum of patients with RA with plasma and serum levels associated with arthritic flare (*82*–*84*). Here, we demonstrate that SPP1^+^MT2A^+^ macrophages are absent in healthy synovium yet enriched in RA, indicating its role as a disease-specific myeloid subpopulation. When comparing myeloid subclusters described in this study to previously published datasets (*85*), myeloid cluster alignment is demonstrated, yet differential myeloid annotation is also observed owing to different experimental approaches and donor heterogeneity across sites. Therefore, it is important to consider these studies together to fully appreciate the diversity of macrophage subsets residing within the synovium in health and disease.

Moreover, we note that inflammatory macrophages residing within the inflamed RA synovium up-regulate multiple NF-κB–related transcription factors. CD40 engagement triggers two distinct NF-κB activation pathways, resulting in the activation of both canonical and noncanonical NF-κB pathways (*86*–*89*). Therefore, targeting CD40-mediated NF-κB activation in RA synovial macrophages has the potential to induce resolution of inflammation.

The pathogenic functions of RA synovial tissue macrophages have an impact on other cells residing within the synovium. Synovial fibroblasts isolated from healthy synovial tissue adapt a pro-inflammatory, invasive phenotype in the presence of CD206^+^CD163^+^ soluble mediators. We further investigated this cross-talk demonstrating that IL-1B^+^CCL20^+^ and SPP1^+^MT2A^+^ synovial macrophage cluster ligands drive an invasive phenotype in synovial fibroblasts with induction of MMPs and collagens. In turn, we demonstrated that fibroblast-derived ligands drive expression of pro-inflammatory gene signatures in SPP1^+^MT2A^+^ synovial tissue macrophages including CD40 receptor expression. This confirms a recent study demonstrating that a distinct subset of HBEGF^+^ macrophages in the RA synovium is capable of promoting synovial fibroblast invasive mechanisms. In addition, this study also reveals that synovial fibroblasts are capable of shaping HBEGF^+^ inflammatory macrophages, thus indicating intricate cross-talk between these two resident synovial cell types (*90*). In contrast, Alivernini *et al.* (*30*) demonstrate that MerTK^+^CD206^+^ synovial tissue macrophages induce repair responses in FLS by secretion of lipid mediators that resolve inflammation. Therefore, it is crucial to consider each of these studies to gain a deeper understanding of the role of synovial tissue macrophages in the pathogenesis of RA.

We demonstrate that CD40-expressing CD206^+^CD163^+^ macrophages are present in IAR of developing RA, before clinical signs or symptoms of disease. CD40-enriched IL-1B^+^CCL20^+^ and SPP1^+^MT2A^+^ macrophages, in synovial tissue, are also a signature present early in disease consistent with two previously published early RA datasets as well as our own cohort (*45*, *51*). Combined, we have demonstrated, using three separate patient cohorts, that the CD40 macrophage signature is an early hallmark of immune dysregulation in RA. Although data on RA preclinical immune dysfunction and the development of autoimmunity into pathogenic arthritis manifestations are lacking, it does remain an area of considerable interest. It is now appreciated that the presence of autoantibodies can precede the clinical onset of RA by several years (*39*). One pilot study of patients with anti-citrullinated protein antibodies (ACPA)–positive arthralgia suggested that macrophage activation may potentially identify subclinical arthritis in patients who subsequently developed RA after 2 years (*91*). This “priming” of myeloid cells before disease is also evident in our recent study, indicating that circulating monocytes from IAR of developing RA display phenotypic features of established RA monocytes and thus are preprogrammed for pro-inflammatory functions (*40*). However, studies of myeloid signatures in at-risk synovium are limited, and tissue biomarkers have not yet been identified. Therefore, this study addresses a gap in our knowledge demonstrating that RA myeloid hallmark signatures are already present in those at risk of developing established disease.

In conclusion, we have presented an extensive characterization of the transcriptional and phenotypic landscape of myeloid populations residing in synovial tissue from health to disease. We describe the presence of a CD40-high myeloid signature that becomes activated early in RA disease pathogenesis, before clinical signs and symptoms, and correlates with disease activity and response to treatment. Uncovering the early molecular patterns and cues that transform this immunoregulatory macrophage population into a dysfunctional inflammatory activation state may provide opportunities to reinstate joint homeostasis in patients with RA.

## MATERIALS AND METHODS

### Experimental design

The objectives of this study were to investigate synovial tissue macrophage populations enriched in the synovial joints of patients with active RA disease and examine their phenotype, transcriptional profiles, and contribution to inflammation. Synovial tissue was obtained from patients with active RA, pre-RA (IAR), and healthy human donors. Synovial fluid and blood from patients with active RA were also collected.

### Patient recruitment, arthroscopies, and sample collection

Patients with active RA are continuously recruited from outpatient clinics at St. Vincent’s University Hospital and The Adelaide and Meath Hospital, Tallaght. Ethical approval to conduct this study was granted by St. Vincent’s Healthcare Group Medical Research and Ethics Committee and the Tallaght University Hospital/St. James’ Hospital Joint Research Committee. Only patients with an actively inflamed knee joint and those that fulfilled the revised ACR/EULAR criteria were included (*2*); demographic and clinical information of patients with RA are presented in Tables 1 and 2. Under local anesthesia, arthroscopies of the inflamed knee were performed using a Wolf 2.7-mm needle. RA synovial tissue biopsies were obtained from the site of inflammation under direct visualization as previously described (*8*). Synovial biopsies from pre-RA (IAR) individuals who are seropositive for ACPA or rheumatoid factor (RF), yet have no clinical signs of inflammation, were also obtained (*40*, *92*). Healthy individuals undergoing arthroscopy during anterior cruciate ligament (ACL) reconstruction surgery were included in this study. Healthy individuals were defined as those who had no evidence of any form of arthritis on history or examination and had no cartilage damage or synovitis on knee arthroscopy. Furthermore, histological examination demonstrated normal synovium with no signs of inflammation. Synovial fluids were also obtained at the time of arthroscopy or at rheumatology clinics, and SFMCs were isolated by Ficoll-Metrizoate density gradient centrifugation (Nycomed, Marlow Buckinghamshire, UK) according to the manufacturer’s instructions.

### Synovial tissue dissociation

Synovial tissue biopsies obtained at the time of arthroscopy were mechanically and enzymatically digested using the GentleMacs dissociator and a soft tumor dissociation kit (Miltenyi Biotec, Germany), according to the manufacturer’s instructions to yield a single-cell suspension of synovial tissue cells. This single synovial cell suspension was then filtrated through a 70-μm cell strainer.

### Flow cytometry

Synovial tissue and SFMC samples were stained using a panel of fluorochrome-conjugated antibodies for flow cytometry. The following antibodies were used in a combination of panels to detect macrophage subsets and activation: CD40, CD45, CD68, CD64, CD163, CD206, CD253, CCR4, CCR7, CXCR1, and CX_3_CR1 (table S1). Cells were resuspended in phosphate-buffered saline (PBS) and incubated with either Live Dead Red or Fixable Viability dye eFluor 450 (Molecular Probes, Thermo Fisher Scientific) for 30 min in the dark at 4°C. Cells were pelleted and resuspended in 100 μl of FACS (fluorescence-activated cell sorting) buffer [Dulbecco’s PBS without Mg^2^^+^ or Ca^2^^+^ (Sigma-Aldrich), 1% heat-inactivated fetal bovine serum (FBS; Biosciences), and 0.05% sodium azide (Sigma-Aldrich), pH 7.4 to 7.6]. To eliminate nonspecific binding of mouse monoclonal antibodies to the Fc-gamma receptor (FcγR), samples were blocked in 5 μl of human FcγR-binding inhibitor for 10 min. Fluorochrome-conjugated antibodies against extracellular markers were then added and samples were vortexed and incubated in the dark at room temperature for 30 min. Part A (100 μl) of the cell fixation and permeabilization kit (Biosciences) was directly added to the fluorochrome mix and incubated for a further 15 min at room temperature in the dark. Cells were then washed, pelleted by centrifugation, and resuspended in 100 μl of permeabilization buffer (part B) containing fluorochrome-conjugated antibodies against intracellular cytokine markers. Following incubation at room temperature for 30 min, samples were washed in PBS and pelleted at 400*g*. Supernatants were removed and the cells were resuspended in 250 μl of PBS. Samples were acquired using the CyAn ADP Flow Cytometer (Backmann Coulter) and the LSRFortessa Flow Cytometer (Beckman Coulter) and analyzed using FlowJo software (Treestar Inc.) and SPICE software (*93*). Cells were gated based on forward and side scatter then dead cells (Fixable Viability dye high) and doublets were removed. Fluorescence Minus One controls were used to determine gating boundaries. To adjust for spectral overlap between detectors, compensation was applied using single stained compensation beads (BD Biosciences). Synovial macrophages were phenotyped based on the expression of CD45^+^ CD68/CD64^+^ and further characterized using the cell surface markers CD40, CD163, CD206, and CD253. The percentage frequency of CD206^+^CD163^+^ macrophages in RA synovial tissue is observed in fig. S1F. Specific macrophage subsets of interest were also sorted using the FACSAria Fusion cell sorter (BD Biosciences) using this gating strategy with total RNA isolated for further RNA-seq (fig. S2A). For SPICE analysis, Boolean gating strategy was adopted to identify all possible cell populations, and this was imported into the SPICE program. Pie charts generated as a result of this analysis represent frequency of coexpression of cell surface markers whereby single marker positive cells are represented by individual arcs surrounding the pie charts, while double, triple, and quadruple costimulatory marker positive cells were represented by overlapping arcs.

### Isolation and culture of monocyte-derived macrophages

Peripheral blood mononuclear cells (PBMCs) were isolated from blood by Ficoll density gradient centrifugation (Lymphoprep; Stemcell Technologies, Canada). A positive selection of CD14^+^ cells was performed by adding MAgnetic Cell Separation (MACS) superparamagnetic microbeads (Miltenyi Biotec, Germany) conjugated with monoclonal anti-human CD14 antibodies to freshly prepared PBMCs in MACS buffer (Miltenyi Biotec), according to the manufacturer’s instructions. Isolated PBMCs were then magnetically sorted and labeled to yield a pure (≥95%) population of CD14^+^ monocytes. CD14^+^ monocytes were seeded at a density of 2 × 10^6^ cells per well in a six-well plate and cultured in RPMI 1640 supplemented with 10% FBS, Hepes (20 mM), penicillin-streptomycin (100 U/ml and 100 μg/ml), amphotericin B (0.25 μg/ml) (all Gibco-BRL, UK), and gentamicin (50 μg/ml; Sigma-Aldrich), supplemented with M-CSF (50 ng/ml) for 8 days to derive in vitro macrophage cultures. Macrophages were then polarized for 24 hours to either M1 macrophages using LPS (100 ng/ml) and IFN-γ (20 ng/ml) or M2 macrophages using IL-4 (20 ng/ml).

### Bulk RNA-seq

CD206^+^CD163^+^ and CD206^−^CD163^−^ macrophages were sorted from RA synovial tissue and fluid using the FACSAria fusion sorter (BD Biosciences), and high-quality RNA was extracted using the RNAqueous total RNA isolation kit (BD Biosciences). High-quality RNA was also isolated from patient-matched monocyte-derived M1 and M2 macrophages using the RNAeasy kit (QIAGEN) and the purity of RNA samples was assessed using the Nanodrop bioanalyzer (Thermo Fisher Scientific). RNA samples were reverse transcribed and sequence libraries were generated using NuGen Ovation Universal RNA-seq System according to the manufacturer’s protocols. The resulting sequencing libraries were analyzed using the Cali-per LabChip GX and quantified using KAPA qPCR. Libraries were normalized and pooled. Each pool was then clustered and sequenced on an Illumina NextSeq500 instrument using 2 × 100-bp paired-end reads, following the manufacturer’s instructions. Raw read quality was evaluated using FastQC before reads were trimmed for adaptors and sequence quality. Trimmed reads were aligned to human CRCh38.84 reference genome using a STAR RNA-seq aligner. Aligned reads were quantified for each gene. Aligned data were evaluated for quality using several quality metrics (e.g., mapping rate, coverage) and visually inspected for samples deviating from the population across multiple metrics and PCA. Statistical analysis of RNA-seq data was performed in R (version 3.5.1). Differential gene analysis was performed with edgeR package to normalize and identify DEGs from counts data. Transcripts with zero counts in more than two-thirds of the samples were discarded from downstream analysis to reduce noise in the expression data. Gene counts were converted to log_2_ counts per million, quantile normalized, and precision weighted. RNA-seq gene features were considered differentially expressed if they satisfied a 2-fold change and false discovery rate (FDR) <0.05 cutoff. FDR control was performed with the Benjamini-Hochberg procedure. Heatmaps and volcano plots were generated in R with the heatmap.2 and EnhancedVolcano functions in the gplots package. Hierarchical clustering was performed with Ward’s linkage. IPA was used to identify canonical pathways overrepresented within DEG.

### Single-cell RNA sequencing

Raw sequencing data from synovial tissue single-cell suspensions were processed using Cell Ranger V3.1 and mapped to the human transcriptome (GRCh38). Single-cell sequencing reads were analyzed with Seurat (V.4.0.3) in R (V4.1) (*94*). Patient samples were individually preprocessed. Genes found in less than three cells were excluded from downstream analysis. Empty droplets were removed using the EmptyDrops function in DropletUtils (*95*). Cell doublets were removed using DoubletFinder (*96*). Cells expressing more than 25% of mitochondrial genes were removed. One RA synovial tissue sample was removed based on high expression of mitochondrial genes, suggesting potentially stressed live cells or a high proportion of dead cells. Data were normalized and scaled, and variable features were identified using the sctransform pipeline in Seurat (*97*). Cell data were integrated using Harmony (*98*). PCA was performed on scaled data. Cell clusters were identified using the FindCluster function in Seurat. Cell clusters were annotated, and macrophages were isolated into a new Seurat object. One healthy control sample was removed due to an absence of macrophages. This resulted in 67,908 macrophages from four RA and four healthy control synovial tissue samples. The macrophages were re-processed using the sctransform pipeline in Seurat to identify macrophage clusters. Clinical information of patients with RA is shown in Table 4. Cell clusters were identified based on literature, and cell cluster markers were calculated using the FindAllMarkers function in Seurat.

DEGs were identified using the FindMarkers function in Seurat using MAST with Bonferroni correction (*99*). DEGs were visualized using EnhancedVolcano (*100*). Pathway analysis using the significantly DEGs was performed using PathfindR (*101*). A phylogenetic tree of cell clusters was built using the BuildClusterTree function in Seurat. Gene modules were calculated using the AddModuleScore function in Seurat. Genes in the oxidative phosphorylation and glycolysis gene modules were curated using the KEGG database. Gene density was calculated using Nebulosa (*102*). Cell interactions were identified using nichenetR (*103*). Receptors, downstream target genes, and potential ligands are identified based on their expression in sender and receiver cells and differential expression between healthy and RA cells. Transcription factor usage in individual cell clusters was estimated using the DoRothEA package (*104*). Gene set variation analysis was performed using the gene set variation analysis (GSVA) package (*105*). DeSeq2 was used to process bulk RNA-seq data from the previously published GSE89408 data (*51*, *106*). GSVA scores for the CD206^+^CD163^+^CD40^+^ module contain the genes CD206, CD163, and CD40. The top 20 cluster markers identified using the FindAllMarkers function in Seurat were used to generate gene modules for the IL-1B^+^CCL20^+^ Cluster and SPP1^+^MT2A^+^ Cluster GSVA scores. Plots were generated with ggplot2, Seurat, and the pathfindR function in R.

### Analysis of the PEAC cohort

The exact methodology of bulk RNA-seq of synovial tissue from 90 individuals with early treatment-naïve RA from the PEAC was described previously (*45*). Total RNA (1 μg per sample) was extracted from whole synovial tissue retrieved from an inflamed peripheral joint using the TRIzol/chloroform method. Bulk RNA-seq (50 million paired-end 75-bp reads per sample) was performed on an Illumina HiSeq2500 platform. RNA-seq data were uploaded to ArrayExpress (accession no. E-MTAB-6141). Data are expressed as regularized log2-transformed reads.

### Cellular bioenergetic function analysis

To assess the metabolic profile of synovial tissue macrophages, we used the noninvasive FLIM technique, which allows for discrimination between glycolysis and oxidative phosphorylation in live cells (*107*–*110*). Following FACS sorting, CD206^+^CD163^+^ and CD206^−^CD163^−^ macrophages were seeded into 18-well 15-μ-Slide (Ibidi) at a density of 1000 cells per well in phenol-free RPMI and left to rest overnight. FLIM was performed using a custom upright (Olympus BX61WI) laser multiphoton microscopy system equipped with a pulsed (80 MHz) titanium:sapphire laser (Chameleon Ultra, Coherent, USA), a water-immersion 25× objective (Olympus, 1.05 numerical aperture), and a temperature-controlled stage at 37°C. Two-photon excitation of NAD(P)H and FAD^+^ fluorescence was performed with 760- and 800-nm excitation wavelength, respectively. A 455/90-nm band-pass filter was used to isolate NAD(P)H fluorescence signal and a 502/47-nm band-pass filter was used for FAD^+^ fluorescence emission. Images (512 × 512 pixels) were acquired with a pixel dwell time of 3.81 μs and 30 s collection time. A PicoHarp 300 TCSPC system operating in the time-tagged mode coupled with a photomultiplier detector assembly (PMA) hybrid detector (PicoQuanT GmbH, Germany) was used for fluorescence decay measurements yielding 256 time bins per pixel. Fluorescence lifetime images with their associated decay curves for NAD(P)H and FAD^+^ were obtained and region-of-interest analysis of the total cells present on the image was performed to remove any background artifact. The NAD(P)H decay curve was generated and was fitted with a double exponential decay without including the instrument response function (Eq. 1).

$$I\left(t\right)=\alpha_{1}e^{\frac{-\tau }{\tau_{1}}}+\alpha_{2}e^{\frac{-\tau }{\tau_{2}}}+C$$ (1)

*I*(*t*) corresponds to the fluorescence intensity measured at time *t* after laser excitation; α_1_ and α_2_ represent the fraction of the overall signal proportion of a short and long lifetime component, respectively. τ_1_ and τ_2_ are the short and long lifetime components, respectively; *C* corresponds to background light. χ^2^ statistical test was used to evaluate the goodness of multi-exponential fit to the raw fluorescence decay data. Here, all the values with χ^2^ < 1.3 were considered as “good” fits. For NAD(P)H, the double exponential decay was used to differentiate between the free (τ_1_) and protein-bound (τ_2_) NAD(P)H. The average fluorescence lifetime was calculated using Eq. 2.

$$\tau_{\text{avg}}=\frac{\left(\tau_{1}\times \alpha_{1}\right)+\left(\tau_{2}\times \alpha_{2}\right)}{\left(\alpha_{1}+\alpha_{2}\right)}$$ (2)

Lifetimes are indicated by τ_1_/τ_2_, and its respective contributions are indicated by α_1_/α_2_. When τ_avg_, is increased, oxidative phosphorylation dominates, and when it is decreased, the cells preferentially use glycolysis. For FAD^+^, only its fluorescence intensity was taken into account. With the addition of NAD(P)H fluorescence intensity, it is possible to calculate the optical redox ratio (ORR) using Eq. 3.

$$\text{ORR}=\frac{{\text{FAD}}^{+}}{\text{NAD}\left(P\right)H}$$ (3)

### Measurement of pro-inflammatory cytokines

To assess the release of cytokines from CD206^+^CD163^+^ synovial tissue macrophages, sorted macrophages were seeded at a density of 30,000 cells per well of a 48-well plate and cultured overnight. Culture supernatants were then collected and protein concentrations of IFN-γ, IL-1β, IL-2, IL-4, IL-6, IL-8, IL-10, IL-12p70, IL-13, and TNFα were measured by multiplex cytokine panel V-PLEX Pro-inflammatory assay; MMP1/3/9 were also measured using the Human MMP 3-Plex Ultra-Sensitive Kit (Meso Scale Discovery, USA) according to the manufacturer’s instructions. Electrochemiluminescence was measured using the MSD Sector Imager 2400.

### Healthy synovial fibroblast functional assays

Healthy synovial fibroblasts were isolated from synovial tissue biopsies from individuals undergoing ACL reconstruction surgery. The same HCFLS donor was used for all experiments for consistency. HCFLS were seeded at a density of 20,000 cells per well into 96-well plates and serum starved for 24 hours in 1% media. Previously stored CD206^+^CD163^+^ synovial tissue macrophages or polarized monocyte-derived macrophage supernatants were added to healthy fibroblasts in a 1:5 ratio and cultured for 24 hours before resulting supernatants were collected for analysis of secreted inflammatory mediators by MSD assay (V-PLEX Pro-inflammatory assay and Human MMP 3-Plex Ultra-Sensitive Kit; Meso Scale Discovery, USA).

### CD40 signaling inhibition assay

CD206^+^CD163^+^ macrophages were FACS sorted from active RA biopsies and seeded at a density of 10,000 cells per well of 96-well plates and left to rest for 2 hours. Macrophages were then cultured in the presence or absence of CD40-TRAF6 inhibitor (CD40-TRAF6 SIGNALING INHIBITOR, 5 μM, Sigma-Aldrich), and resulting supernatants were collected the following day for future MSD assays.

### Statistical analysis

Statistical analyses were performed using Prism 9 software. Wilcoxon signed rank test or Mann–Whitney was used for analysis of nonparametric data. Student’s *t* test was used for parametric data. *P* values of less than 0.05 (*P < 0.05) were determined as statistically significant.
