# Supplementary material for: Loss of synovial tissue macrophage homeostasis precedes rheumatoid arthritis clinical onset
Source: Sci Adv. 2024 Sep 25;10(39):eadj1252. doi: 10.1126/sciadv.adj1252 (PMC11423874; doi:10.1126/sciadv.adj1252)
Supplement: Supplementary file 1 — Figs. S1 to S10 Table S1 [file sciadv.adj1252_sm.pdf]

Science Advances
AAAS

# Supplementary Materials for

## Loss of synovial tissue macrophage homeostasis precedes rheumatoid arthritis clinical onset

Megan M. Hanlon *et al.*

Corresponding author: Ursula Fearon, fearonu@tcd.ie; Megan M. Hanlon, hanlonme@tcd.ie

*Sci. Adv.* **10**, eadj1252 (2024)
DOI: 10.1126/sciadv.adj1252

**This PDF file includes:**

Figs. S1 to S10
Table S1

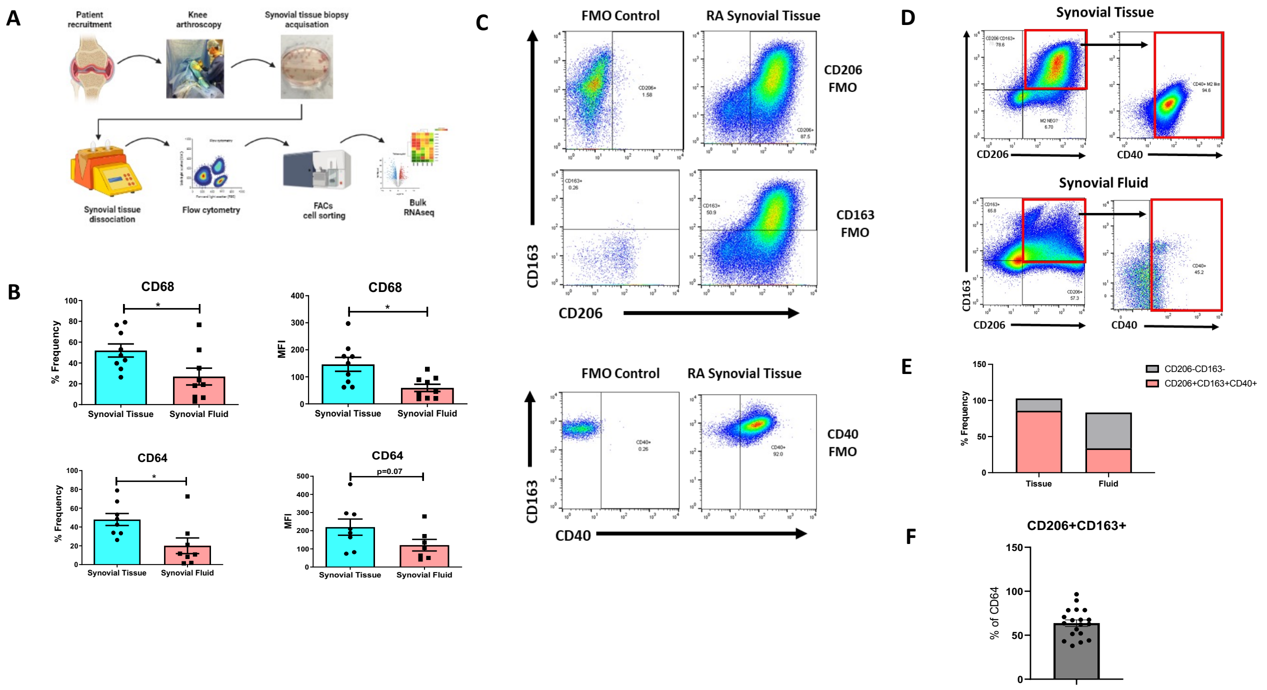

**Fig. S1: Synovial macrophage gating strategy. (A)** Schematic of workflow overview created using Biorender.com. Synovial tissue was acquired at the site of inflammation during knee arthroscopy of active RA patients. Synovial tissue was dissociated, and macrophages profiled using flow cytometry, cytometric cell sorting and bulk RNAseq. **(B)** Percentage frequency and median fluorescence intensity (MFI) of pan macrophage markers CD68 and CD64 in RA synovial tissue (n=8-9) compared to RA synovial fluid (n=7-9). Gates set according to FMO controls. Data represented as mean ± SEM with each symbol representing a different sample. Statistical analysis was performed using Mann Whitney U test *p<0.05 significantly different from synovial tissue. **(C)** Representative flow cytometric plots indicting CD206, CD163 and CD40 gating using FMO controls pre-gated on CD45+CD64+ macrophages. **(D)** Representative dot plots representing CD206$^{+}$CD163$^{+}$ subset and subsequent CD40 expression in RA synovial tissue and fluid. **(E)** Relative proportion bar chart representing CD206$^{+}$CD163$^{+}$CD40+ and CD206-CD163- macrophage subsets in RA synovial tissue vs fluid. **(F)** Bar chart representing percentage frequency of CD206+CD163+ macrophages in RA synovial tissue (n=19).

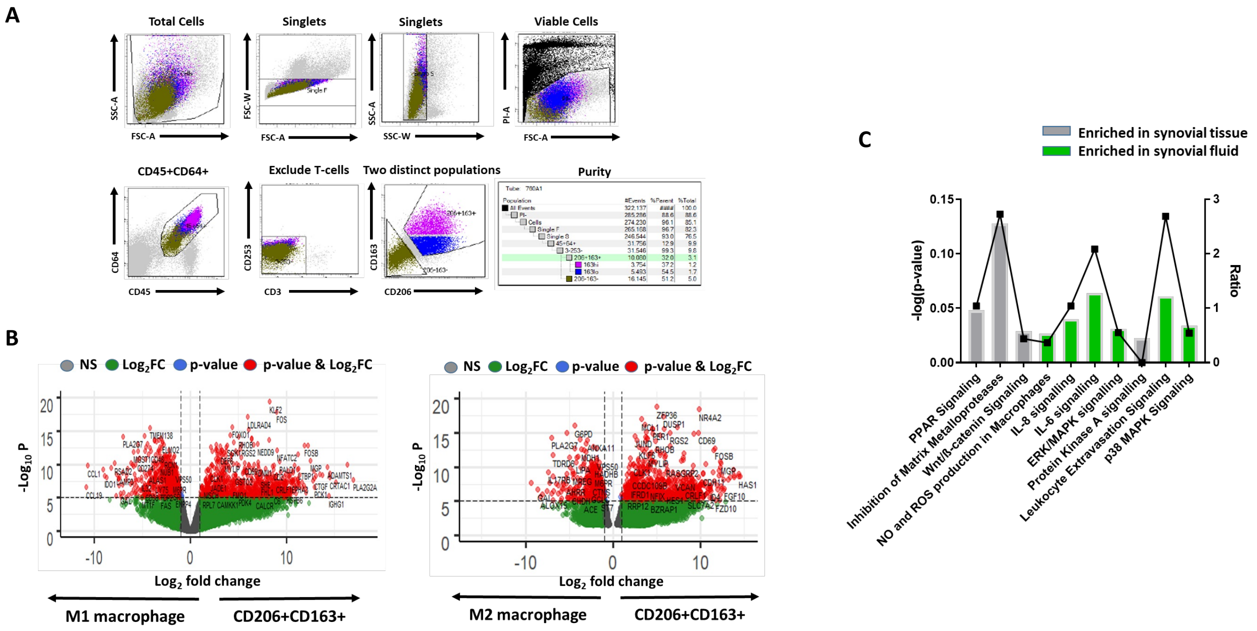


**Fig. S2: Bulk RNA-seq macrophage transcriptional profiling. (A)** Gating strategy to identify and sort CD206$^+$CD163$^+$ and CD206$^-$CD163$^-$ macrophages from RA synovial tissue and synovial fluid mononuclear cells using the FACS Aria Fusion sorter. Cell purity was also assessed using the FACSAria Fusion sorter. **(B)** Volcano plots of the relative difference in expression of DEGs between CD206$^+$CD163$^+$ synovial tissue macrophages and RA monocyte derived M1 and M2 macrophages. **(C)** Ingenuity pathway analysis (IPA) of enriched pathways between CD206$^+$CD163$^+$ synovial tissue and fluid macrophages. Ratio represents the number of genes from the list that maps to the pathways divided by the total number of genes that map to the same pathway.

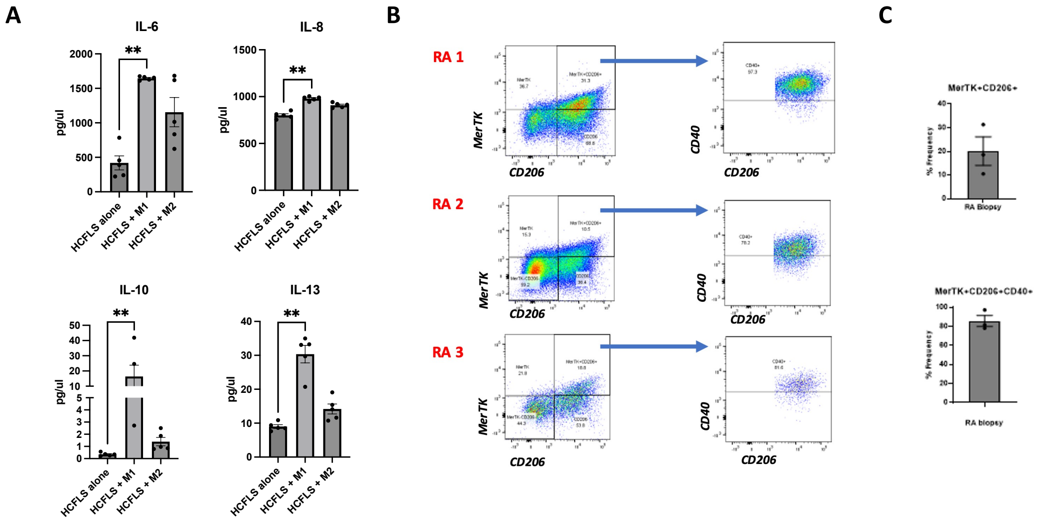


**Fig. S3: Functional analysis of synovial tissue macrophages. (A)** Dot plots of cytokine expression following addition of RA M1 and M2 macrophage conditioned media on healthy synovial fibroblasts (n=5). Data expressed as mean ± SEM using one-way ANOVA with Tukeys multiple comparisons test, **p<0.01 significantly different from each other. **(B)** Representative flow cytometric dot plots of 3 independent RA synovial tissue samples demonstrating the frequency of MerTK+CD206+ macrophages and co-expression of CD40. **(C)** Bar graphs indicating the percentage frequency of MerTK+CD206+ and MerTK+CD206+CD40+ macrophages in RA synovial tissue.

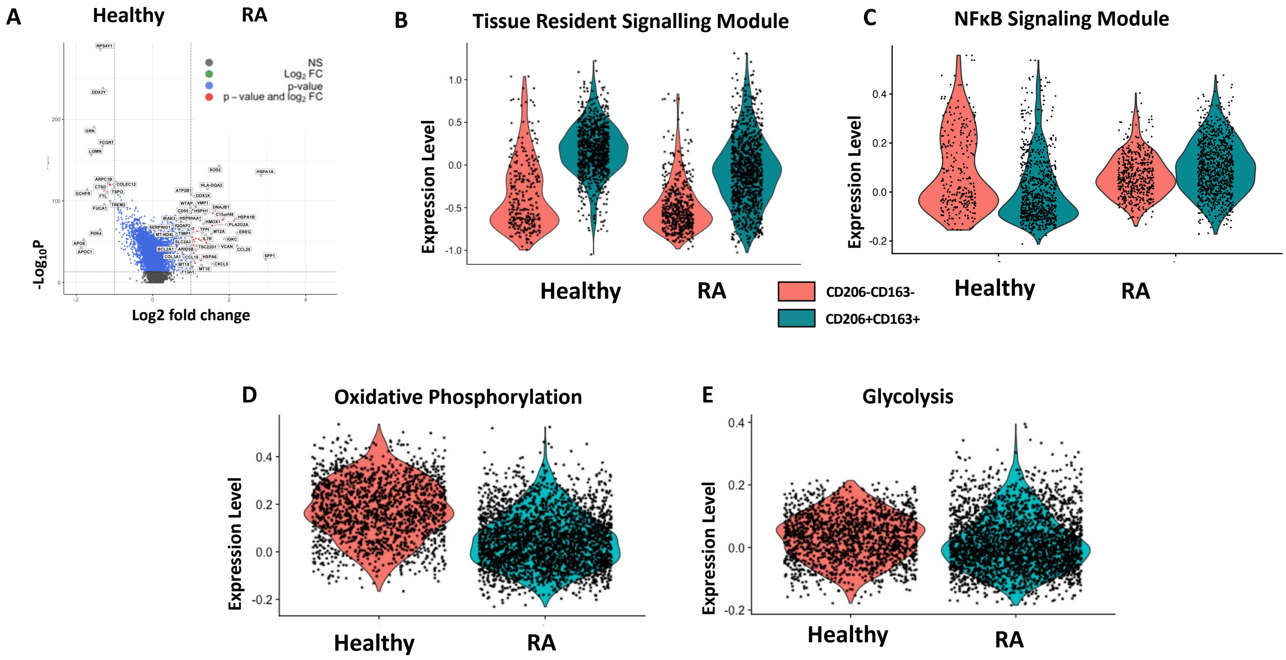


**Fig. S4: scRNA-seq analysis of synovial tissue macrophages. (A)** Volcano plot depicting differentially expressed genes between RA and healthy control synovial tissue macrophages. Violin plots indicating expression level of **(B)** tissue resident gene module, **(C)** NFκB signalling module, **(D)** oxidative phosphorylation and **(E)** glycolysis gene modules in RA and healthy control synovial tissue macrophages.

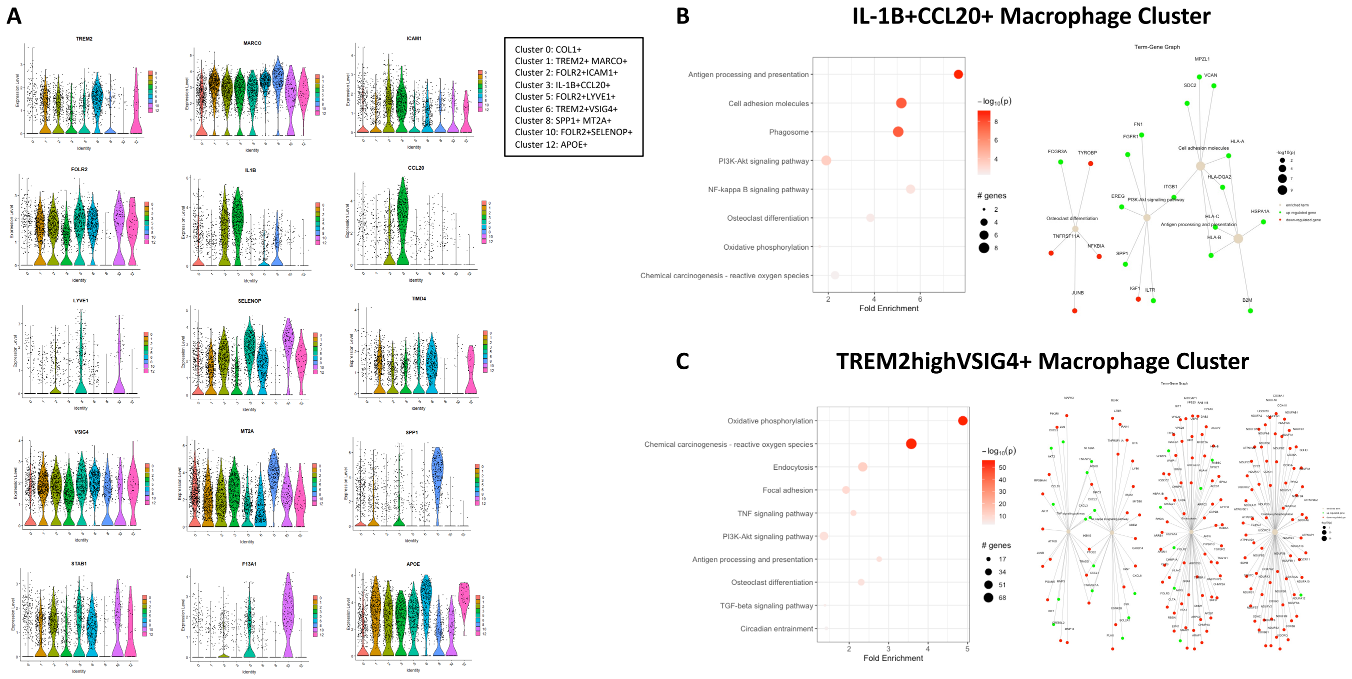


**Fig. S5: scRNA-seq synovial tissue macrophage transcriptional profiling. (A)** Violin plots representing log-normalized expression values of synovial tissue macrophage cluster markers. Analysis of pathways enriched in **(B)** IL-1B+CCL20+ and **(C)** TREM2highVSIG4+ macrophages, colour intensity represents significance and dot size the number of genes within each pathway that are differentially expressed. Term plot of indicated pathways with significant enrichment in **(B)** IL-1B+CCL20+ and **(C)** TREM2highVSIG4+ macrophages. Colour indicates up or downregulation of specific genes within the pathway and dot size represents statistical significance of change.

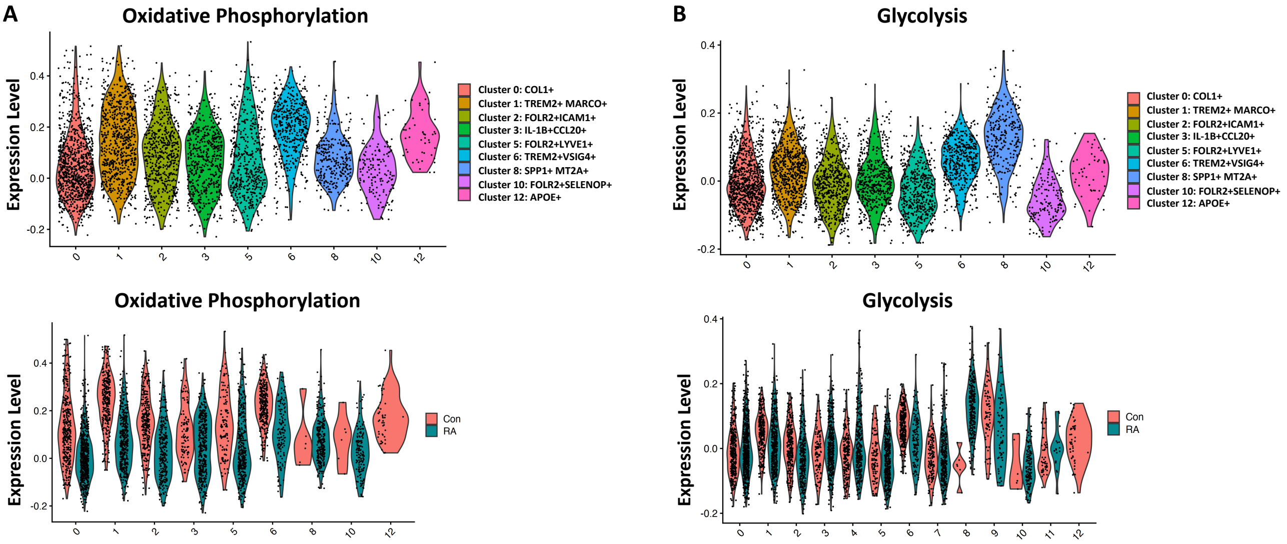


**Fig. S6: scRNAseq analysis of metabolism associated gene modules in synovial tissue macrophages.** Violin plots indicating expression level of **(A)** oxidative phosphorylation and **(B)** glycolysis gene modules in identified synovial tissue macrophage clusters in RA and healthy control individuals.

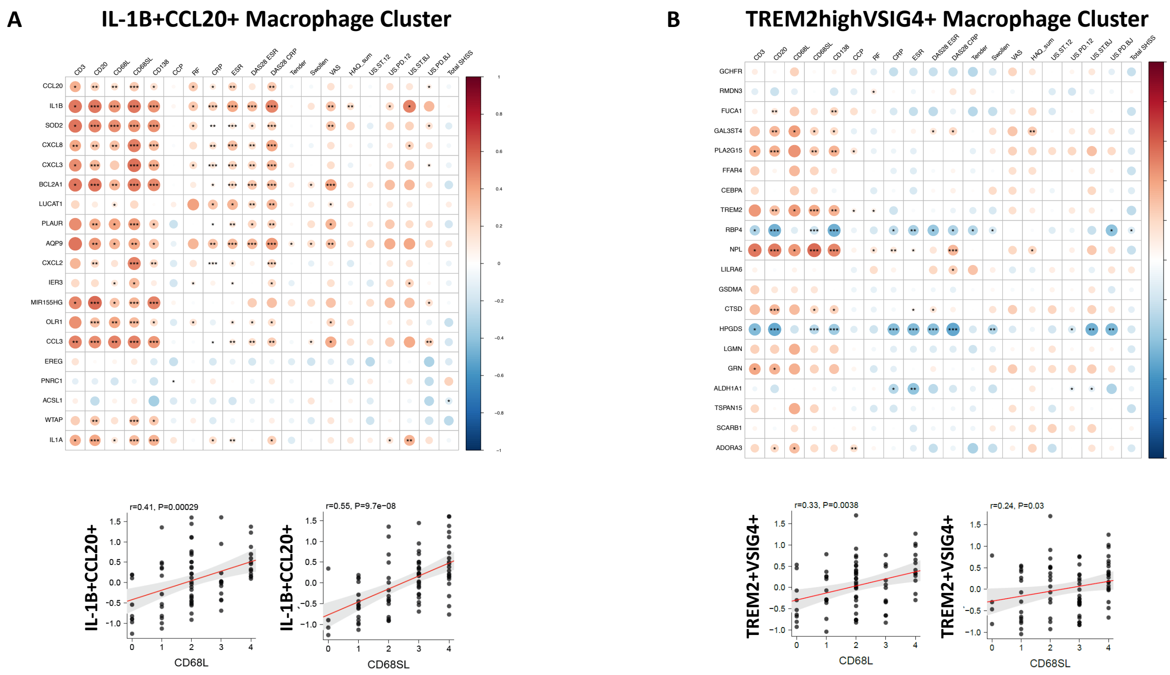


**Fig. S7: Synovial tissue macrophage cluster correlations using the PEAC cohort.** Correlation heatmap showing Spearman correlation of **(A)** IL-1B+CCL20+ and **(B)** TREM2highVSIG4+ macrophages gene modules against baseline immune and clinical parameters in the PEAC cohort (n=90). (CCP, anti-cyclic citrullinated peptide antibody titer; RF, rheumatoid factor titer; CRP, C-reactive protein; ESR, erythrocyte sedimentation rate; DAS, disease activity score; VAS, visual analog score; HAQ, health assessment questionnaire), ultrasonographic scores (ST, synovial thickness; PD, power doppler) at the biopsy joint (Ultrasound ST/PD BJ) or across 12 representative joints (Ultrasound ST/PD 12) and radiographic parameters (Total Sharp van der Heijde score). Two-tailed Spearman's correlation between synovial expression of **(A)** IL-1B+CCL20+ and **(B)** TREM2highVSIG4+synovial tissue macrophage clusters with CD68L and CD68SL in the PEAC cohort (n=90).

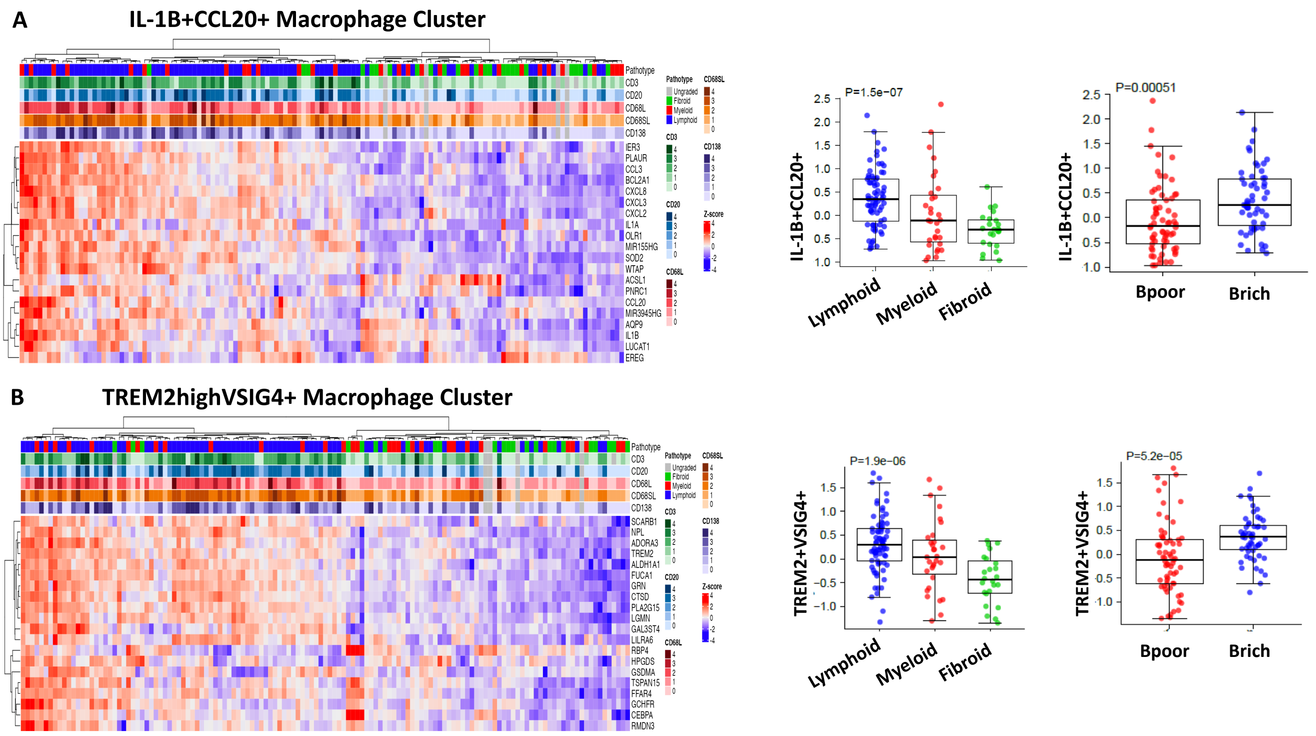


**Fig. S8: Synovial tissue macrophage cluster correlations with synovial pathotypes.** Heatmap of 2,964 RNA-seq genes differentially expressed between three histological pathotypes (lympho-myeloid, diffuse-myeloid, and pauci-immune fibroid) (FDR < 0.05, n = 87). Upper tracks show histological scores for CD3, CD20, CD68L, CD68SL, and CD138 and overall pathotype in **(A)** IL-1B+CCL20+ and **(B)** TREM2+VSIG4+ macrophage clusters. **(C)** Cell-specific gene scores compared across histology pathotypes in **(A)** IL-1B+CCL20+ and **(B)** TREM2+VSIG4+. Statistical analysis by one-way ANOVA with Bonferroni post-test.

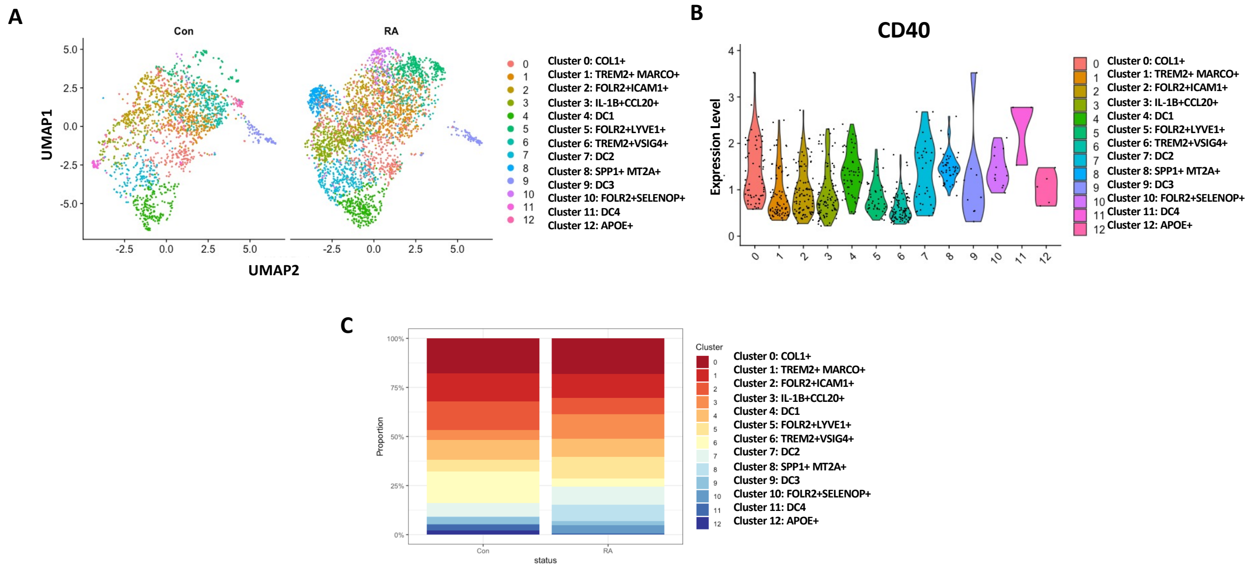


**Fig. S9: Synovial tissue myeloid clusters in health and disease. (A)** UMAP depicting synovial tissue myeloid cluster distribution including four DC clusters between RA and healthy synovium. **(B)** Violin plot representing CD40 gene expression levels across all synovial tissue myeloid clusters. (C) Proportional cluster abundance between established RA patients and healthy control individuals across myeloid clusters.

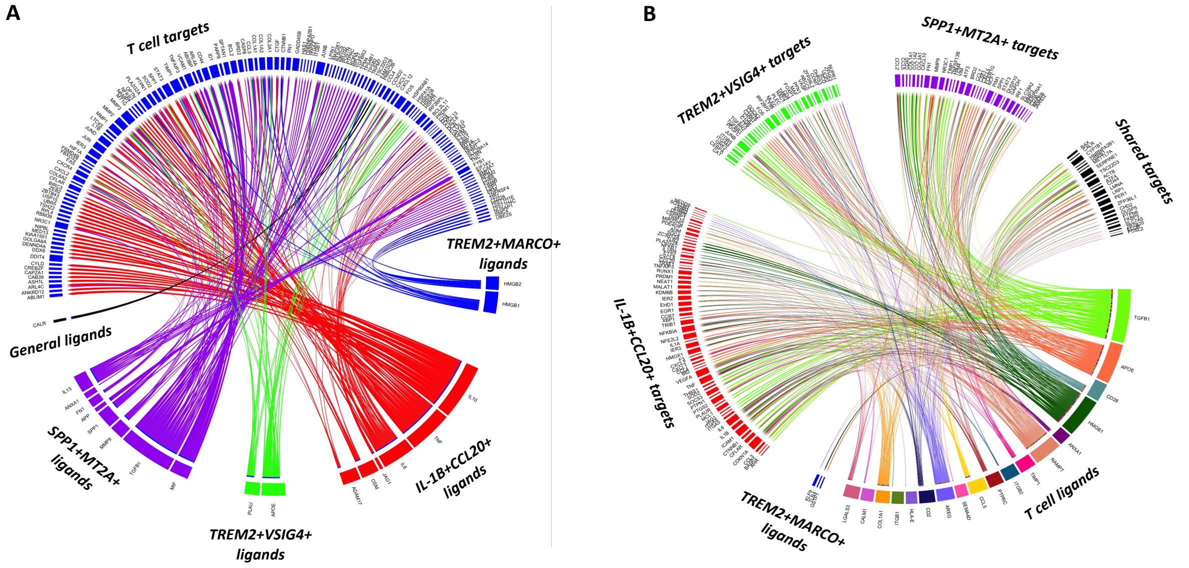


**Fig. S10: Synovial tissue myeloid-T cell crosstalk. (A)** and **(B)** Circo plots depicting the top predicted receptor and ligand interactions between specific indicated macrophage clusters and synovial T cells.

| Antibody | Fluorochrome | Clone |
|---|---|---|
| **CD3** | **APC** | **OKT3** |
| **CD40** | **APC-CY7** | **5C3** |
| **CD45** | **BV510** | **H130** |
| **CD64** | **PerCPCY5.5** | **10.1** |
| **CD68*** | **PE-CY7** | **Y1/82A** |
| **CD163** | **FITC** | **GHI/61** |
| **CD206** | **PE** | **19.2** |
| **CD253** | **APC** | **RIK-2** |
| **CCR4** | **BV605** | **L291H4** |
| **CCR7** | **PeCF594** | **GO43H7** |
| **CXCR1** | **PE-CY7** | **8FI** |
| **CXCR3** | **BV650** | **GO25H7** |
| **CX$_3$CR1** | **APC** | **2A9-1** |

**Table S1: Panel of fluorochrome antibodies used for flow cytometry analysis.** Fluorochrome antibodies used in this study (* indicates intra-cellular stain).
